# Senolytics as Modulators of Critical Signaling Pathways: a Promising Strategy to Combat Brain Aging and Neurodegenerative Disorders

**DOI:** 10.1007/s12035-025-05504-1

**Published:** 2025-12-06

**Authors:** Ishika Singh, Abhishek Kumar Singh

**Affiliations:** Department of Biotherapeutics Research, Manipal Academy of Higher Education, Manipal, 576104 Karnataka India

**Keywords:** Autophagy, Brain aging, Neurodegenerative diseases, Senescent cells, Senolytics

## Abstract

Aging of the brain, an intricate process, is a significant risk factor for neurodegenerative disorders (NDDs), such as Alzheimer’s disease and Parkinson’s disease. Senescent cell accumulation is an important hallmark of brain aging. These cells resist apoptotic cell death, produce proinflammatory cytokines, increase oxidative stress, and store toxic proteins that exacerbate neurodegeneration. These senescent cells cause neuroinflammation and dysfunction of the neuronal microenvironment by transmitting senescent phenotypes to neighboring healthy cells. Senolytics have become a viable treatment option to reduce the effects of brain aging since they specifically target and destroy senescent cells. Numerous senolytic compounds, such as dasatinib, fisetin, and quercetin, effectively eliminate senescent cells and reduce the accumulation of harmful substances, including misfolded toxic protein aggregates and reactive oxygen species, thereby helping to maintain tissue homeostasis. These medications aid in reducing oxidative stress and inflammation, two significant factors in brain aging and NDDs, by encouraging the removal of senescent cells. The key molecules involved in this process are mTOR, Nrf2-Keap1, AMPK, and Sirtuin 1 (SIRT1). The modulation of the mTOR and AMPK pathways affects autophagy and cellular metabolism, facilitating the elimination of harmful accumulations and damaged cell organelles. In addition, cellular repair and improved antioxidant defense are encouraged by the activation of the SIRT1 and Nrf2 pathways. The combination of senolytic therapy with these signaling pathways provides a novel approach to attack the cellular and molecular foundations of brain aging and neurodegenerative disorders.

**Graphical Abstract:**

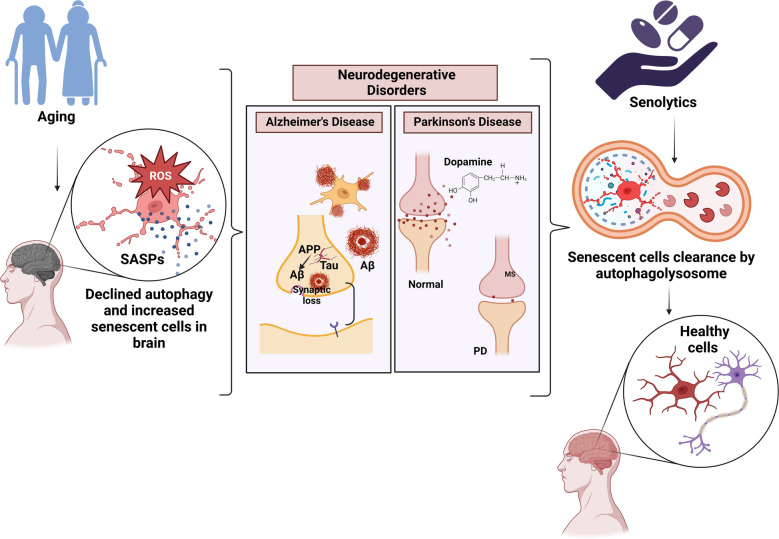

## Introduction

Aging is a natural process associated with structural and functional deterioration, affecting the ability to respond to environmental stressors. Aging affects mainly the brain, causing its functional capability to decline over time. Impairments in learning and retention, time for decision-making, attention, perception of sensory information, and motor function constitute typical manifestations of age-associated cognitive decline. There are several hallmarks of aging, including senescence, proteotoxicity, oxidative stress, and decreased autophagy.

Cellular senescence is a state of cell cycle arrest used as a protective mechanism by which cells avert the spread of injured cells and the development of malignancies. However, it may also stimulate aging-related characteristics and diseases. Cells undergoing senescence exhibit decreased autophagy and increased accumulation of toxins inside the cells, which results in the progression of numerous neurodegenerative diseases (NDDs). NDDs are linked to multiple attributes, such as inflammation, increased levels of oxidative stress, dysfunctional mitochondria, and misfolded or damaged protein aggregation inside the cell. Another intriguing feature of senescent cells is that they can transfer these senescent characteristics to neighboring cells (paracrine senescence) through the secretion of molecules called senescence-associated secretory phenotypes (SASPs), which include chemokines, cytokines, and tumor necrosis factor (TNF). SASPs can activate the senescent cell antiapoptotic pathway (SCAP), leading to the inhibition of apoptosis in aged cells. As a result, the cells fail to clear out waste accumulation and develop neurotoxicity.

Senopathies impair the nervous system’s ability to operate and maintain its structure. Some NDDs associated with aging are Alzheimer’s disease (AD), Parkinson’s disease (PD), and amyotrophic lateral sclerosis (ALS), the incidence of which has increased annually. The two most prevalent NDDs, AD and PD, usually tend to develop in the aged population, with an increase in risk with age. A tissue analysis of patients suffering from AD revealed deposits of senile plaques and abnormalities in tau proteins. Senile plaques are formed because of the deposition of amyloid β peptides, and the hyperphosphorylation of tau is responsible for their abnormal characteristics. Since neurons lack the tendency to undergo cell division like other cells of the body, this deposition increases over time and hinders the formation of a neuronal signaling network.

Hence, clearing senescent cells (SnCs) is a promising alternative for alleviating neurological disorders such as AD and PD. The group of compounds that selectively target and induce cell death in senescent cells is known as senolytics. As a result, they may encourage an extended lifespan and health span and postpone, stop, or even reverse senescence. Senolytics target different SCAPs to selectively kill senescent cells via autophagy. The activation of sirtuin, AMPK, and Nrf2-Keap1 and the inhibition of mTOR by senolytics are the major pathways associated with the induction of autophagy to clear SnC. In addition to inhibitors belonging to the Bcl-2 family, several other molecules categorized in different classes of senolytics selectively cause the death of SnC through a cascade of signaling pathways. Recently, mitoTAMs, which particularly target mitochondria with increased ΔΨ_m_, were shown to exhibit characteristics of senolytics, suggesting another class of senolytics that can be categorized as senolytics with mitochondrial targeting ability. Research has shown that senolytic treatment led to improved spatial memory in 4-month-old male and female mice treated with a combination of quercetin and dasatinib or with fisetin [1].

**Fig. 1:**
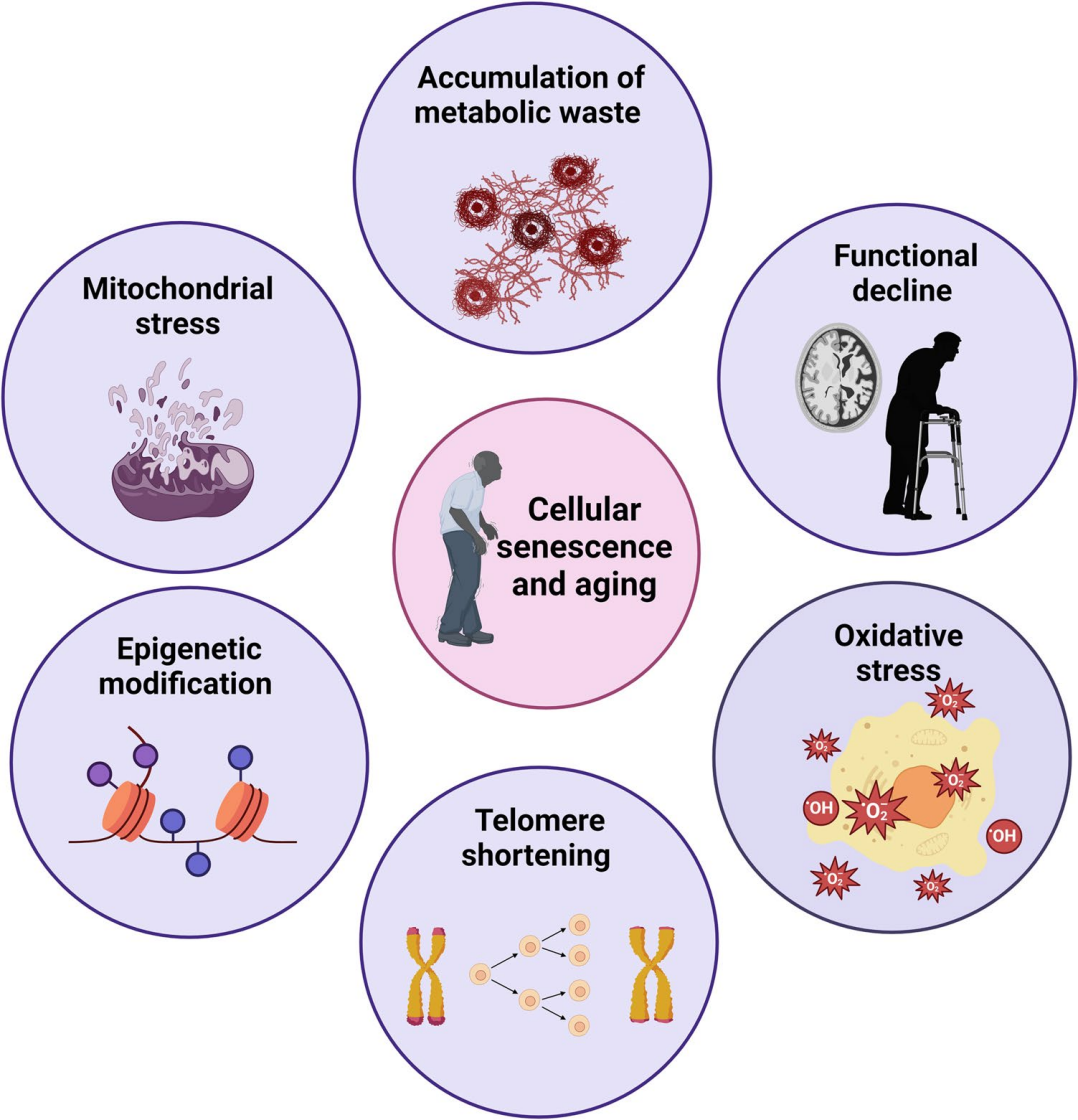
Cellular senescence is characterized by mitochondrial dysfunction, epigenetic modifications, telomere shortening, increased production of reactive oxygen species (ROS) and oxidative stress, accumulation of toxic waste due to autophagy inhibition, and functional decline, as observed in patients suffering from various neurodegenerative disorders

The biomedical community and researchers have worked hard over the past 20 years to create novel therapeutics for NDDs, but despite their efforts, there has been no cure. For example, the only drugs available for the treatment of AD are those that provide only symptomatic relief from the condition rather than curing the root cause. This review focuses on senolytics as potential therapeutic targets for alleviating the progression of various NDDs (Fig. 1).

## Cellular Senescence

**Fig. 2:**
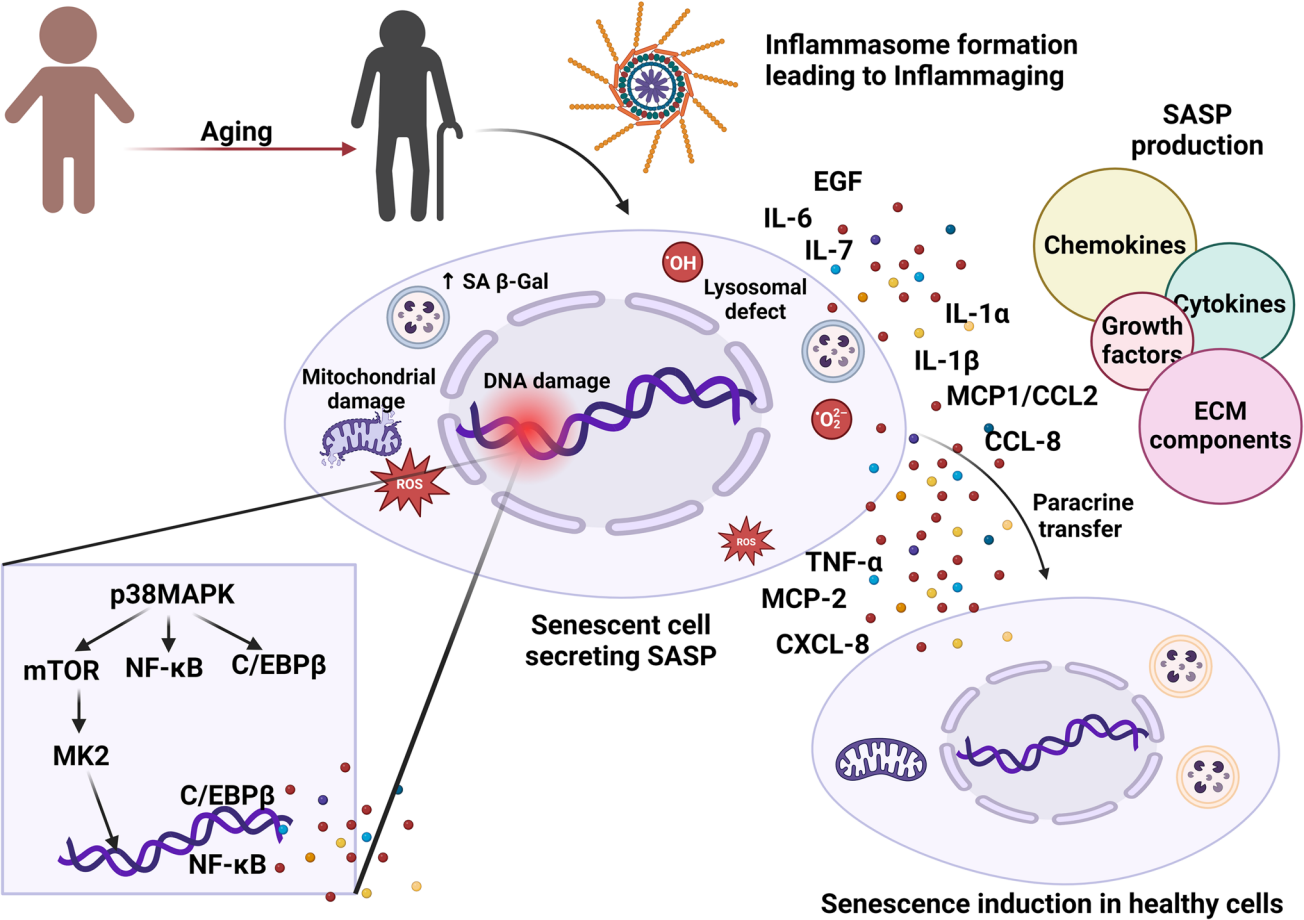
Senescence, inflammation, and SASP. Age-associated increases in inflammaging lead to increased incidences of age-associated neurodegenerative disorders via the upregulation of proinflammatory cytokines and other senescence-associated secretory phenotypes (SASPs), such as chemokines, growth factors, and extracellular matrix (ECM) components

Aging is a phenomenon characterized by a significant reduction in the ability of a cell to maintain homeostasis, which ultimately contributes to oxidative and mitochondrial stress. It also leads to metabolic impairments and escalates DNA damage. Cellular senescence is a prominent hallmark of aging and refers to the irreversible arrest of the cell cycle [2]. It is a state of cell division arrest where cells are not able to proliferate, even in the presence of optimal stimuli for growth and division [3]. This phenomenon is closely linked with the upregulation of cyclin-dependent kinase inhibitors such as *CDKN2A*/p16^*Ink4a*^ and *CDKN1A*/p21^*CIP1/WAF1*^. These cells are associated with the release of SASPs, such as chemokines, cytokines, and certain growth factors, in the immediate environment. SASP spreads in a paracrine manner, transmitting senescent phenotypes to neighboring healthy cells. They also trigger the senescent cell anti-apoptotic pathway, which protects these cells from cell death-associated apoptotic pathways [4]. In 1961, Hayflick and Moorhead were the pioneers in identifying the process of cellular senescence when they subcultured human fibroblasts in series and described it as a feature attributed to intrinsic factors [5]. The cell cycle arrest at growth phases 1 and 2 of cell division is a distinguishing feature of cellular senescence, which differs from quiescent cell division arrest, which typically occurs during the G0 phase. Additionally, cells in the quiescent stage of the cell cycle can resume cell division upon the reversal of unfavorable conditions; however, once they reach senescence, the cells cannot resume division [6, 7]. SnCs tend to accumulate with time in the body, contributing to increased demand for energy and resulting in altered lipid metabolism. Several studies have also revealed that these cells contribute to the aggregation of iron, which can be linked to age-associated diseases. The cellular senescence can be initiated by multiple stimuli, including DNA damage. [8], dysfunctional mitochondria [9], oxidative stress [10], and other factors are secreted by senescent-associated secretory proteins (SASPs) (Fig. 2).

### DNA Damage

Double-stranded breaks in DNA are less common, with an average of 10–50/day. However, despite being less common, they contribute to the loss of genetic information and are thus highly mutagenic, leading to toxicity. The two prominent types of machinery responsible for repairing this damage are homologous recombination and nonhomologous end joining. Homologous recombination is mostly prevalent during the late G2 and S phases of the cell cycle, whereas nonhomologous end-joining repair is prevalent during all phases of the cell cycle, including G1 [11]. In mature neurons, the nonhomologous mechanism is the main DNA damage repair pathway; however, the homologous mechanism is highly active in other dividing cells of the nervous system [8].

ATM, a serine/threonine protein kinase, is highly upregulated during double-stranded DNA damage. Activated ATM kinase phosphorylates and activates checkpoint kinase 2 (CHK2) and p53, both of which play essential roles in the induction of senescence [12]. A cascade of cellular pathways, known as DNA damage responses (DDRs), is activated by DNA damage. Double-strand breaks are the major elicitors of such responses. Apart from their direct action to restore the integrity of the DNA, they also participate in the excitation of other cellular responses, including checkpoints in the cell cycle, protein turnover, and gene expression. The main regulatory molecules of such responses are p53/p21 and p16/p16INK4a-pRB. Appropriate regulation is crucial since decreased action of DDRs can lead to the activation of cellular senescence, whereas the overactivation of DDRs is detrimental and can be a consequence of age-related NDDs. Shortening of telomeres as a part of the repeated replication of cells is also a contributing factor to the activation of DDRs and initiation of the process of senescence in cells [13]. The brain is very sensitive to cellular senescence, as it comprises mostly non-proliferative cells, making them vulnerable to defective DNA repair. This can result in the aggregation of unrepaired DNA lesions. This largely contains non-proliferative neuronal cells and is therefore particularly vulnerable to defective DNA repair, which can lead to the accumulation of unrepaired DNA lesions. These DNA lesions have been proposed to be the cause of the neuropathology observed in several NDDs [14].

#### Mitochondrial Dysfunction

Cellular senescence is caused by dysfunctional mitochondria, which can be explained by a decrease in respiratory ability per mitochondrion as well as a decrease in the mitochondrial membrane potential, along with an increase in the concentration of reactive oxygen species (ROS) [9]. While a slight decrease in the mitochondrial membrane potential can lead to ROS production and drive the electron transport chain (ETC) toward a more oxidized state, a prolonged decrease in the MMP can result in the complete failure of the ETC. Apart from this decreased mitochondrial membrane potential, it can increase the likelihood of proton leakage.

The decline in respiratory capacity has diverse conclusions as far as the role of individual ETC complexes is concerned. For example, complexes III and IV prominently lose function with increasing age in the heart muscle, whereas complex I seems to be more sensitive to loss of function with age in the brain, skeletal muscle, and liver [15].

As the largest ETC complex, the functions of complex I and the production of ROS are attributed to assembly fidelity, which decreases with age, whereas knocking down only one complex I assembly factor is enough to trigger cellular senescence [16]. As this complex in the ETC is associated with the oxidation of the reduced state of nicotinamide adenine dinucleotide (NADH) to NAD^+^, the decline in respiratory ability may result in the disturbance of the NAD^+^/NADH balance observed during aging and cellular senescence. In contrast, during senescence induced by oncogenes, the NAD^+^ salvage pathway is positively regulated, thus contributing to increased SASP production through the suppression of the AMPK signaling pathway [17].

Mitochondrial dysfunction resulting from a decrease in either sirtuins, such as SIRT3 or SIRT5, the expansion of mtDNA, or the administration of an inhibitor of the ETC, such as rotenone or antimycin A, led to a reduction in the cytosolic NAD^+^/NADH ratio and led to the arrest of senescent cell growth. SIRT3 is a mitochondrial deacetylase that removes acetyl groups from enzymes and components of the ETC. On the other hand, SIRT5 plays a role in the succinylation and glutarylation of ETS components. A decrease in either SIRT3 or SIRT5 is associated with a reduction in functional enzymes of ETS, lower ATP production, and elevated production of ROS. Excessive production of ROS leads to oxidative damage, increased mitochondrial permeability, and structural and functional collapse of mitochondria [18]. Similarly, tissue samples collected from PolG^D257A^ mice with major mtDNA mutations [19] exhibit extensive senescence as a consequence of reduced NAD^+^/NADH ratios. Excitingly, in each of these instances, the resulting senescence was quantified by an SASP in the absence of its proinflammatory arm, including the activation of NF-κB and overproduction of interleukins (ILs), such as IL1, IL6, and IL8 [20].

#### Oxidative Stress

Senescent cells are characterized by dysregulated metabolic functions, including deteriorated lysosomal function and accumulation of lipofuscin, an aggregate of intracellular catabolism that is not biodegradable [21]. The improper functioning of lysosomes decreases mitophagy, causing the aggregation of distorted mitochondria, which results in increased ROS. This leads to the targeting of lysosomes and aggravates macromolecular damage, leading to increased expression of senescence phenotypes [22].

Reactive oxygen species are prominent and major regulators of senescence in cells and trigger all the associated senescence phenotypes mentioned above. Free radicals, such as HO and RO, are highly reactive and short-lived because they usually target molecules in proximity to their generation site. Elevated levels of peroxides (oxidative stress), single- or double-strand breaks, and DNA base modifications are triggered by labile iron, and there is oxidative damage to proteins and lipid peroxidation. All these effects have the potential to initiate and elicit senescence in cells.

## Senescence-Associated Secretory Phenotype

The extracellular environment of a cell comprises a variety of molecules that it secretes over its lifetime. However, the secretome of senescent cells varies drastically from that of their normal proliferating counterparts. SASPs can be categorized into cytokines, chemokines, growth factors, proteases, and insoluble extracellular matrix (ECM) factors.

The SASPs identified in neurons vary and differ based on the stage of senescence, cell type, model organism, and molecules that initiate senescence. One of the prominent cytokines that play a major role in SASPs is interleukin-6 (IL6). IL6 is a proinflammatory cytokine that is directly regulated by the DNA damage signaling pathways ATM (ataxia-telangiectasia mutated) and CHK2 (checkpoint kinase 2). Another notable cytokine is IL1 (α and β), which is overexpressed in senescent cells. Normal brain aging and age-associated NDs are linked with microglia-mediated increases in the levels of IL-1β and IL-6, which are proinflammatory cytokines [23]. Senescent cells also overexpress chemokines such as CXCL-8, along with GROα and GROβ. The components of the CCL family that are generally increased in senescent cells include MCP-1, 2, and −4 (CCL-2, −8, and −13); HCC-4 (CCL-16); eotaxin-3 (CCL-26); and macrophage inflammatory protein (MIP)−3α and −1α (CCL-3, −20) [24]. C–C motif chemokine ligand 2 (CCL2) is the prominent SASP molecule involved in neuronal senescence [25].

Brain aging is a natural and intricate process linked with an increased number of senescent cells that induce neuroinflammation and a decline in immunity. To acknowledge this crucial relationship between aging and inflammation, inflammaging was introduced as an emerging concept. The process of inflammaging refers to elevated levels of markers linked with inflammation, such as chemokines, cytokines, and growth factors, eventually leading to chronic damage. Given the prominent role of inflammation in aging, it has now been accepted as a prominent hallmark [26].

Even though the population of senescent cells is very low in tissue compared with that of healthy cells, senescent cells are still capable of generating age-associated NDDs [27]. The secretion of SASPs from senescent cells helps accelerate the aging process via the disruption of cellular homeostasis and the transmission of senescent phenotypes to neighboring healthy cells through the paracrine transfer of SASPs [28]. The SASPs include the production of diverse molecules, among which IL-1 *α*/*β*, IL-6, IL-8, TGF-*β*, and TNF-*α* are the most studied and are considered to be the major SASPs [29]. The secretion of these proinflammatory molecules facilitates the recruitment of immune system cells to the site of senescent cell accumulation, resulting in their degradation and leading to inflammation.

**Fig. 3:**
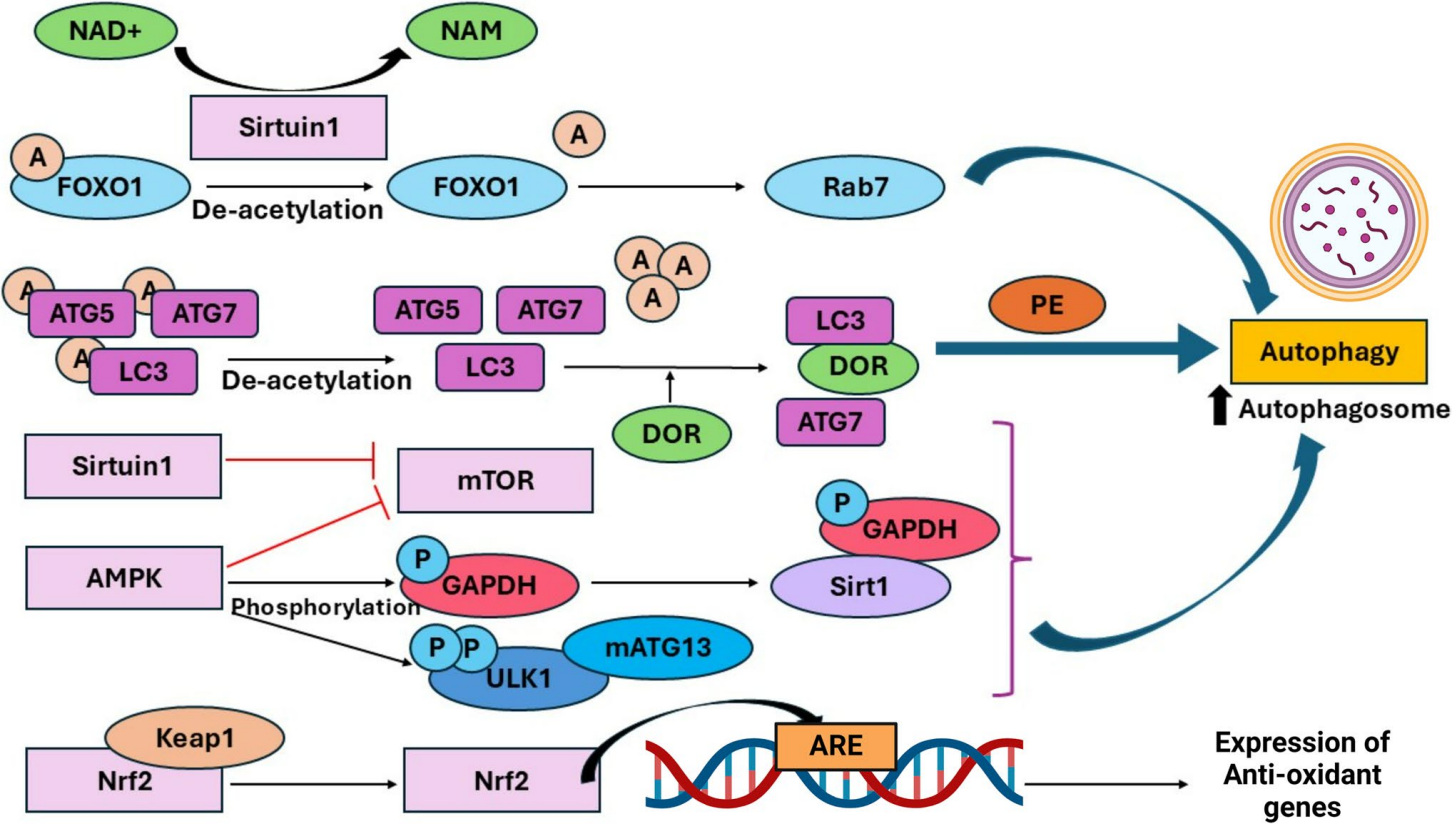
Pathways associated with autophagy regulation. Sirtuin 1, AMPK, mTOR, and Nrf2Keap1 are crucial metabolic pathways associated with the modulation of autophagy. Sirtuin 1 facilitates the deacetylation of FOXO1, which further leads to Rab7-mediated activation of autophagy. It also interacts with several autophagy genes (Atgs) and LC3, leading to their deacetylation and facilitating autophagosome formation. Sirtuin1 and AMPK also act as direct inhibitors of mTOR, thus activating autophagy. AMPK is also associated with GAPDH and ULK1, which are crucial for the development of autophagosomes. Nrf2 is released from Keap1 and migrates to the nucleus, where it binds to AREs, leading to the transcription of genes involved in autophagy

During aging, inflammaging is particularly detrimental to health because of its continuous elicitation of the immune system over a long period. During inflammaging, the receptors associated with differentiating self-cells from non-self-cells fail to perform their functions. As a result, the innate immune system is overexcited and starts attacking the host’s self-cells [30]. Research conducted to deduce the association between inflammation and SASPs revealed that S6 kinase 1 (S6K1), a ribosomal kinase, led to a substantial decrease in the release of SASPs such as IL-1β. S6K1 is a crucial regulator of mTOR [31] (Fig. 3). S6K1 is activated via phosphorylation of its threonine 389 residue by the mTORC1 complex. Upon activation, it enhances the production of IL-1β by regulating the protein synthesis machinery. In addition, S6K1 is associated with a feedback loop in the mTOR pathway, which helps to increase the levels of S6K1 and IL-1β. In the brain, increased production of S6K1 is associated with chronic inflammation. Studies have revealed that knocking out the S6K1 gene in mice leads to decreased production of IL-1β and protects them from metabolic disorders induced by inflammation [31].

## Senescence in Brain Aging and Neurodegenerative Diseases

Although brain aging is a prevailing and natural phenomenon associated with every individual, the extent of this process vastly varies among individuals, emphasizing heterogeneity in the process of brain aging. This heterogeneity is a result of intricate correlations among various factors, such as genetic factors, lifestyle habits, and diseases. These factors either lead to the aggravation of neuropathy or protection against age-associated neuropathic diseases [32]. Divergences in the predicted brain age from the chronological age are regarded as signs linked with changes in brain function and structure due to aging and are termed brain aging.

Brain atrophy (reduction in the volume of gray matter and thinning of the lining of the cortex) [33], a decline in the integrity and volume of white matter in the brain, a reduction in white matter integrity and volume, and dysregulation of information transfer across synapses are major changes that accompany brain aging [34, 35]. These phenotypes, when drastic, can be regarded as early signs of accelerated aging or an underlying cause of neurodegenerative disease. Although senescence associated with other tissues has recently been associated with aging and several disorders have developed due to aging, its intricacy in brain aging has just begun to be studied. However, increasing evidence indicates that senescence might also influence brain aging, as previously demonstrated for other organs.

Among the total cells in the brain, glial cells account for approximately 50% of all cells and play crucial roles in modulating the process of brain homeostasis in the context of health and disease [36]. When exposed to oxidative stress or inhibitors associated with the function of the proteasome, murine and human astrocytes display a decreased ability to proliferate as well as altered expression levels of several well-recognized markers of cellular senescence, such as the activity of SA-β-gal and the levels of the proteins p53, p21^WAF1/CIP1^, and p16^INK4A^ [37]. Evans et al. reported that human astrocytes tend to undergo both telomere erosion-independent and p53-dependent senescence. In that group, the frontal cortex was observed to have elevated p16^INK4A^ expression in aged and AD groups in comparison to that in younger subjects [38]. The process of senescence can also be triggered in astrocytes via the deposition of amyloid-β plaques, which initiates the activity of SA-β-gal, p16^INK4A^ expression, and a SASP response linked with the kinase p38 MAP [39].

Senescent glial cells exhibit an increased presence of effectors associated with functional microglia. Elevated expression of proinflammatory cytokines, such as IL-1β, TNF-α, and IL-6, has been reported in aged microglia [40]*.* However, only studies based on flow cytometry analysis revealed an increase in the number of p16^INK4a−^ and p21^WAF1/CIP^-positive microglia derived from the brains of aged mice. This inclination correlated with elevated autofluorescence (senescent cell characteristics because of lipofuscin aggregation) and increased γH2AX and Bcl-2 (antiapoptotic proteins) levels [41].

Once the neurons mature, they have the potential to last for the entirety of our lives and exhibit significant plasticity potential. This allows mature neurons to regulate complex networks of synapses and continuously adapt to changing environmental conditions. Although a drastic decline in neuronal function occurs with age, efforts to target neuronal senescence for the treatment of neurodegenerative diseases are still lacking [42]. Given that neurons exist as postmitotic, nonproliferating cells (arrested in the cell cycle G0 phase), during neuronal senescence, they must adhere to other mechanisms apart from the inhibition of proliferation, such as those adopted by other postmitotic cells. The most widely used marker of cellular senescence is the increased activity of SA-β-gal, although it is not very specific. Studies have revealed increased levels of SA-β-gal in the hippocampus region of the brains of aged rats [43], cultured cells for an extended duration [44], and neurons from cerebellar granules [45]. To ensure senescence, the neuronal cultures were also characterized by increased levels of SAPS markers in vitro. In long-term cultures of hippocampal neurons, several markers associated with cellular senescence were found to be upregulated in response to the Cdk inhibitor p16^INK4A^ (Cdkn2a), which increased the stimulation of kinase p38 MAP and decreased lamin B1, resulting in changes in chromatin structure.

### Alzheimer’s Disease

Alzheimer’s disease (AD) is the most prominent neurodegenerative disorder and was first described by Alois Alzheimer in 1906. It is regarded as the most common form of dementia in older adults, accounting for 60–70% of all dementia cases [46]. It is manifested by three prominent hallmarks: Aβ plaques, tau phosphorylation, disorientation, and memory impairment. Amyloid β (Aβ) plaques are deposited due to the cleavage of amyloid precursor protein (APP) by β- and γ-secretase, generating the Aβ peptide. This fragment leads to toxicity in neurons either directly by causing neuroinflammation or indirectly by inducing the production of free radicals. Another pathological hallmark of AD is the hyperphosphorylation of tau proteins, which are involved in microtubule formation and are primarily distributed in axons. AD progression is categorized into 3 phases: (1) presymptomatic, in which there is no cognitive decline; (2) mild cognitive impairment (MCI), associated with short-term memory decline due to the loss of neurons in the hippocampal region of the brain (lasting 2–7 years); and (3) AD, including both short- and long-term memory deterioration (lasting 3–8 years, leading to a more aggressive stage and death). Presently, there is no cure for AD, and the available medications provide relief from only the symptoms.

The most prominent risk factor for AD is aging and associated senescence. Previously, therapeutics used to combat AD have focused primarily on its pathological hallmarks. However, in recent years, research has shifted toward strategies for combating the effects of cellular senescence for the prevention and treatment of this disease. Astrocytes and microglia undergoing senescence are prominently observed in the brains of patients with AD, along with increased activity of SA-β-gal [47]. By inducing the NF-κB and p38/MAPK pathways, TNF-α and inflammatory cytokines such as IL-6 and IL-8 are secreted by these senescent cells, which also transfer these characteristics to neighboring cells via paracrine transfer [48]. Microglia have been shown to undergo senescence in patients with AD, which is attributed to the shortening of telomeres [49]. This senescence leads to the formation of SASPs in AD conditions and the aggregation of senescent cells, which is associated with the formation of reactive oxygen species and pro-inflammatory molecules. It also promotes the accumulation of amyloid plaques and the hyperphosphorylation of tau proteins [50]. A study conducted on mouse models of AD revealed that markers associated with cellular damage and cell cycle arrest, including p16, p21, and p53, as well as markers associated with inflammation, such as IL-6 and TNF-α, were found to be overexpressed [51].

#### Parkinson’s Disease

PD is the most prominent ND after AD and is characterized by the loss of functional neurons in the pars compacta region of the substantia nigra, including the deposition of α-synuclein aggregates. The primary symptoms of PD include tremors, muscle stiffness, balance impairment, and a decreased ability to perform spontaneous and automatic actions. The Global Burden of Disease Study estimates that the number of PD cases will double from approximately 7 million in 2015 to approximately 13 million in 2040, suggesting a potential ‘PD pandemic’ [52]. Increased expression of SA-β-gal and an abundance of senescent cells in brain tissue from Parkinson’s disease patients are linked to α-synuclein deposition. In PD brain tissues, markers associated with senescent cells, such as p16^INK4a^, and diverse SASP molecules, such as MMP-3, IL-6, 1α, and 8, increase. These findings imply that cellular senescence is involved in dopaminergic neurodegeneration [53].

Increased expression of SA-β-gal and an abundance of senescent cells in brain tissue from Parkinson’s disease patients are linked to α-synuclein deposition. In PD brain tissues, the levels of p16INK4a and various SASP factors, such as MMP-3, IL-6, 1α, and 8, increase. These findings imply that senescence is involved in the degeneration of dopaminergic neurons [53]. Mitochondrial dysfunction is strongly implicated in the pathophysiology of PD. A mitochondrion-associated kinase protein, phosphatase, and tensin homolog (PTEN)-induced kinase 1 (PINK1), was identified in patients with early-onset PD. It has also been shown to be deposited in dysfunctional mitochondria [54].

#### Multiple Sclerosis

Multiple sclerosis (MS) is categorized as an autoimmune disorder in which the body’s immune system starts attacking the myelin sheath, which covers the neurons, causing their degeneration and dysregulation of neurotransmission. The term MS refers to the specific regions deposited by scar tissue (plaques or lesions) because of an attack by immune cells. MS can be distinguished into two phases, where phase 1 is marked by relapses and remissions, and phase 2, wherein a more critical accumulation of disability occurs. Aging and age-associated senescence are common drivers of MS. The accumulation of SnC with aging and the secretion of SASPs, such as cytokines, are secreted by senescent microglia, resulting in NDs such as MS. Furthermore, demyelinated white matter lesions contain lipofuscin + aged glial cells and neurons derived from MS patients but not usually white matter. Finally, the remyelination ability of brain progenitor cells is limited by cell senescence, which accelerates the development of multiple sclerosis [55].

A study was conducted to evaluate the difference in the expression of p16^INK4a^, a gene associated with cellular senescence, in patients with MS and healthy volunteers. The study included 52 MS patients and 38 healthy individuals. Those authors reported that patients above the age of 50 and suffering from MS had higher median p16^INK4a^ expression than did controls [56]. Another study revealed that senescence phenotypes are upregulated in microglia and macrophages post-demyelination and reduced during remyelination in young mice. However, senescent phenotypes are prevalent in demyelinated lesions, leading to ineffective remyelination. Markers of SASPs, such as CCL11, were found to be elevated in lesions of aged mice, suggesting that targeting SnCs can prove to be a beneficial therapeutic option for MS treatment [57].

#### Amyotrophic Lateral Sclerosis

Amyotrophic lateral sclerosis (ALS) is a progressive neurodegenerative disease that affects motor neurons. It is associated with muscular weakness, failure of respiration, and degeneration of motor neurons, eventually leading to death. To date, the cause of the disease is unknown; however, mechanisms such as oxidative stress, mitochondrial dysfunction, gene mutation, neuroinflammation, and dysfunction of synaptic transmission are considered contributing factors to ALS. The presence of two prominent variants, *TDP-43* and *FUS*, in the cytoplasm, which are found in the nucleus under normal conditions, is thought to be responsible for neurodegeneration. Mutation of an important lncRNA, *C9ORF72*, which is responsible for autophagy, is another prominent contributing factor to ALS [58].

Age is a well-known causative factor for ALS, with close associations with cellular senescence. However, the exact mechanism by which senescence affects ALS is not well understood. A study was conducted to elucidate the expression of senescence markers during the loss of motor neurons and gliosis in the spinal cord and how they lead to the progression of paralysis in a SOD1^G93A^ rat model of ALS. They observed that microglia, motor neurons, astrocytes, and the lumbar spinal cord all presented positive results for p16 staining [59]. Another study revealed that the expression of the p16 and p21 markers of senescence in the frontal cortex of the ALS brain is associated with the senescence of astrocytes and disruption of the normal cell cycle in the early phase of the disease. They reported that p16 and p21 are present in neurons, glial cells, and astrocytes in the brain tissues of patients collected post-mortem, suffering from ALS, compared with control tissues [60].

To date, how SnCs potentially contribute to the pathological symptoms of ALS is unclear, and this connection needs to be extensively studied. However, evidence suggests that the presence of SnCs and associated markers in ALS patients and disease models reflects an overlap in these two conditions. Further elucidation of the role of SnCs in ALS can help develop novel therapeutics to combat this disease.

### Signaling Pathways Associated with Brain Aging and Diseases

#### mTOR Signaling

Mammalian target of rapamycin (mTOR) is the major pathway that links oxidative stress and autophagy. It plays a crucial role as an energy sensor molecule. mTOR is composed of two associated protein complexes, namely mTORC1 (comprising 5 subunits: mTOR, raptor, mLST8, PRAS40, and Deptor) and mTORC2 (comprising Deptor, Rictor, mLST8, mSIN1, and Protor-1). Studies have shown that mTORC1 is associated with autophagy and that its inhibition leads to increased longevity. Under normal conditions, when nutrients are available, mTOR is activated, and it phosphorylates the ULK1 complex, thus inactivating it and the process of autophagosome formation. However, during nutrient deprivation, mTOR is inhibited, which leads to autophosphorylation and activation of ULK1 and the formation of autophagosomes, which decrease toxic accumulation in the cell.

Rapamycin is a natural molecule produced by a soil bacterium, *Streptomyces hygroscopicus*, which was first identified as an antifungal antibiotic in 1975 on Easter Island (Rapa Nui), from which it was named. Rapamycin can act on mTORC1 by directly blocking the recruitment of substrate, thus inhibiting it and restricting access to active sites by binding the FRB domain of the immunophilin FKBP12 associated with TOR.

### AMPK Signaling

Another sensor molecule is AMPK, which senses variations in cellular energy, such as deviations in the levels of ATP and AMP. This protein comprises β and γ, which are regulatory subunits, and α, which is a catalytic subunit. AMPK is activated when ATP levels decrease through the phosphorylation of threonine in the α subunit. It can regulate cell growth and proliferation as well as autophagy; hence, its signaling is thought to be associated with aging and lifespan [61].

AMPK directly or indirectly influences the autophagy process. It directly functions by binding to the mTORC1 receptor, thus rendering it incapable of phosphorylating ULK1 and inhibiting it. The direct pathway is capable of directly phosphorylating ULK1 to initiate the process of autophagosome formation. Moreover, AMPK signaling is involved in the activation of gene transcription associated with the regulation of oxidative stress via the phosphorylation and activation of FOXO at its regulatory sites.

Metformin also hinders the transfer of phosphate groups and aggregation of α-synuclein to safeguard against mitochondrial dysfunction, oxidative stress, and the regulation of autophagy, and decreases neurodegeneration and neuroinflammation through the activation of the AMPK signaling pathway [62].

#### Sirtuin 1 Signaling

Another molecule associated with delaying cellular senescence is sirtuin, which was discovered in the 1970s. From Sirtuin 1 (SIRT1) to SIRT7, there are seven isoforms of sirtuin, among which SIRT1 plays a crucial role as a deacetylase whose activity is regulated by NAD^+^ [63]. SIRT1 is activated in response to the presence of NAD^+^ and the deacetylase FOXO1, which helps Rab7-mediated increases in the transcription of genes associated with autophagy activation. Sirtuin 1 is associated with the deacetylation of genes (atg5 and atg7), which are involved in autophagy and crucial for the formation of autophagosomes. LC3 deacetylation is also facilitated by Sirtuin 1, which subsequently interacts with DOR, a nuclear protein, facilitating their migration from the nucleus to the autophagosome. Atg7 also interacts with deacetylated LC3, leading to its conjugation to phosphatidyl ethanolamine (PE) and further integration with the early membrane of the autophagosome.

#### Nrf2-Keap1 Signaling

The nuclear element factor is linked to erythroid 2, an essential factor for controlling oxidative stress in the Nrf2-Keap1 pathway, which is linked to Kelch-like ECH-associated protein 1 (Ji et al., 2015). Under normal circumstances, Nrf2 is linked to Keap1 in the cytoplasm, preventing its activation and mediating its destruction. However, in stressful situations, such as when there is an increase in reactive oxygen species (ROS), Nrf2 can separate from Keap1 and move into the nucleus. It interacts with autophagy response elements (AREs) present in the promoter area of the antioxidant gene transcriptional unit within the nucleus to aid in transcription [64].

## Interplay Among the mTOR, AMPK, SIRT1, and Nrf2-Keap1 Signaling Pathways

Signaling pathways such as the mTOR, AMPK, SIRT1, and Nrf2-Keap1 pathways are responsible for the modulation of complex biological phenomena such as aging. They regulate processes such as senescence, autophagy, and neuroinflammation, which in turn contribute to age-associated neurodegenerative diseases. Various studies have revealed a close interconnection between these processes, leading to synergistic action in the context of protection from NDDs. Activation of the mTOR pathway is responsible for the growth of cells; however, its chronic activation can lead to senescence and declined autophagy in cells. Studies in neuronal and glial cell models have revealed that mTOR activation leads to overproduction of SASPs, such as IL-1β, IL-6, and MMPs, which are inflammatory markers, suggesting that long-term mTOR activation leads to neuroinflammation [65].

Another study on the effect of mTOR inhibition via rapamycin in an APP/PS1 mouse model of AD revealed that treatment with rapamycin inhibited mTOR, which consequently led to a decrease in the expression of SASP markers and increased expression of autophagy markers. The cognitive function of rapamycin-treated mice was also found to improve [66]. AMPK is another pathway that is closely linked with autophagy regulation and mTOR signaling. A study conducted on in vitro astrocyte cultures treated with 5-aminoimidazole-4-carboxamide riboside, an AMPK activator, revealed reduced p16 and β-galactosidase levels as well as improved mitochondrial function [67]. Similarly, another study demonstrated that the use of metformin results in activation of the AMPK pathway in aged and AD mouse models. They also revealed that activation of AMPK decreased microglial activation and facilitated neurogenesis in mice, highlighting that AMPK activation is closely related to neuroinflammation and brain aging [68]. The influence of SIRT1 on cognition has been studied, revealing that increased expression of SIRT1 leads to improved cognitive function and decreased expression of markers associated with neuroinflammation in a mouse model [69].

Moreover, the overexpression of SASP markers such as MMP-2 and IL-6 in Aβ-treated in vitro models, because of astrocyte senescence, also led to the loss of neuronal function and synaptic integrity. These findings suggest that a wide variety of cells are affected in the brain by senescence and that they transmit these characteristics to neighboring healthy cells [39]. In addition, various studies have reported that a decline in autophagic flux in neuronal and glial cells in both in vitro and in vivo models leads to oxidative stress and mitochondrial damage, which promotes the p53-p21 pathway, leading to neuroinflammation [70]. Studies have also shown that p62, an important autophagy component that binds cargos to the autophagosome, is linked with Nrf2 dissociation from Keap1. p62 binds to keap1, thus facilitating the dissociation of Nrf2. Once dissociated, Nrf2 enters the nucleus and promotes the transcription of AREs [71]. Hence, these mechanisms are interconnected, and disruption of one pathway closely affects the functions of other pathways. Therefore, therapeutic interventions such as senotherapeutics influencing multiple pathways could be potential alternatives for the treatment of NDDs.

## Long Noncoding RNAs

Noncoding RNAs with nucleotide lengths greater than 200 are recognized as long noncoding RNAs (lncRNAs) and have been shown to play a significant role in neurodegenerative diseases. Earlier research highlighted the participation of lncRNAs in various pathways involved in the aging and growth of cells, both of which are closely linked to autophagy. Additionally, the negative regulation of these lncRNAs can contribute to the progression of many neurological diseases [71].

A previous study revealed that the level of LC3B, which is an important marker of autophagosome formation, was elevated in an Aβ-treated AD model in SH-SY5Y cells when lncRNA17A was knocked down compared with that in cells with elevated expression of lncRNA17A. This was associated with the up- and downregulation of the GABABR2 receptor, which plays a role in the inhibition of transmission across the synapse, with up- and downregulation of lncRNA 17A. This study revealed that decreased levels of lncRNA 17 A might help regulate autophagy via alternative splicing of GABABR2 and thus help in the elimination of senescent cells [72]. Another study conducted on an APP/PS1 mouse model of AD revealed that the expression of the lncRNA NEAT1 increases with age. The lncRNA NEAT1 leads to the ubiquitination of PINK1, PTEN-induced putative kinase 1 (PTEN-kinase 1), which is important for mitochondrial function. Hence, its ubiquitination and mitophagy can lead to mitochondrial dysfunction and toxic waste accumulation [73].

Like in AD, the lncRNA NEAT1 also plays a crucial role in PD via the regulation of PINK1 and LC3B [74]. Another study revealed that the negative regulation of the lncRNA SNHG1 is associated with mTOR inhibition and the activation of autophagy, which promotes the survival of dopaminergic neurons in patients with PD. However, another lncRNA, HAGLROS, has been shown to regulate the autophagic mechanism via regulation of the PI3K/AKT/mTOR pathway [75].

Another important lncRNA is NEAT1_2, which is overexpressed in neurons associated with motor activities in patients with ALS compared with healthy individuals. This highlights its potential for use as a disease marker in determining ALS [76]. Moreover, lncRNAs also play a significant role in the progression of HD. Various lncRNAs, such as MEG3, NEAT1, and XIST, are involved in HD development by associating with miRNAs and decreasing their ability to bind to their target mRNAs [77].

## MicroRNAs and Their Association with Senescence

MicroRNAs (miRNAs) are small noncoding molecules with a length of approximately 22 bp and have recently gained attention for their role in regulating cell death via the modulation of pathways associated with cellular senescence at the transcriptional and posttranscriptional levels [78]. Recent studies have indicated the ability of miRNAs to modulate aging and senescence in cells either through the degradation of mRNAs or via translational downregulation [79]. This process is referred to as posttranscriptional gene silencing, wherein miRNAs form partial bonds with the 3’UTRs of target mRNAs, eventually hindering their translation into their respective proteins and leading to a decrease in mRNA stability [80].

Numerous miRNAs, such as miRNA-9, miR-22, miR-29, miR-34a, miR-210, miR-494, miR-106b, miR-125b, miR-126, miR-146a, miR-21, miR-449a, the miR-17–92 cluster, and the miR-200 family, are differentially expressed in senescent cells [81]. There is growing evidence that suggests the involvement of miRNAs in signaling cascades involved in crosstalk with the cell cycle and senescence through crucial p16 and p53 signaling pathways and SASP regulators [78].

Under stress conditions, activation of the p16 pathway can lead to the induction of senescence, consequently leading to CDK inhibition, thereby inhibiting the phosphorylation of retinoblastoma, deactivating E2F, and inducing senescence. Many miRNAs directly modulate the process of cell senescence through interaction with p16 mRNA and regulating its expression levels. For example, miRNA-24 (miR-24) suppresses the expression of p16 via a posttranscriptional approach by attaching to the 3′*UTR* of p16 mRNA in human diploid fibroblasts, thus inhibiting replicative senescence. In addition, various other miRNAs regulate the process of cellular senescence via the activation of the p16 signaling cascade without attaching directly to p16. For example, suppression of DNA methyltransferase 1 (DNMT1) expression directly by miR-217 leads to the upregulation of both p16 protein and mRNA expression, eventually resulting in human skin fibroblast senescence [78]. Like p16 signaling, various miRNAs, such as miR-25, miR-30d, and miR-125b, inhibit senescence by binding directly to p53 and inhibiting its transcription (Kumar et al., 2011). In contrast, other miRNAs are indirectly involved in the process of regulating senescence via the regulation of p53 [82].

A diverse group of miRNAs modulates the levels of SIRT1. Notably, miR-34a induced cellular senescence by targeting SIRT1 in various tissues. miR-217 was found to induce a premature senescence-like phenotype through direct targeting of SIRT1. miR-34a overexpression can induce senescence, and miR-34a is considered a promising target for treating inflammation and age-related diseases [83]. It has also been reported that targeting genes in antioxidant pathways other than SIRT1 contributes to oxidative stress-mediated cellular senescence. However, another study reported that increased expression of miR-34a can lead to increased levels of IL-6, a crucial SASP [84].

Deletion of another miRNA, miR-155, leads to decreased expression of IL-1β, a proinflammatory cytokine [85]. miR-9 is known to regulate the proinflammatory effect of IL-17A [86]. An increase in the expression of miR-335 significantly diminishes the expression of inflammatory molecules, including IL-1β, −6, and −8 [87].

miRNAs are responsible for the regulation of an important cell cycle regulator, p16, a gene related to tumor suppression. p16 works by acting as an inhibitor of cyclin-dependent kinase (CDKs), especially CDK4 and CDK6. These CDKs are important for the cell transition from the G1 to S phase of the cell cycle. The presence of p16 inhibits this transition and leads to cell cycle arrest, contributing to senescence. miRNAs such as miRNA-24, miRNA-300, miRNA-514, miRNA-613 and miRNA-141 help the cell transition from G1 to S phase by inhibiting or suppressing p16 expression. miRNA-24 binds to p16 and causes a decrease in its translation, whereas the other miRNAs lead to suppression of p16 expression.

**Fig. 4:**
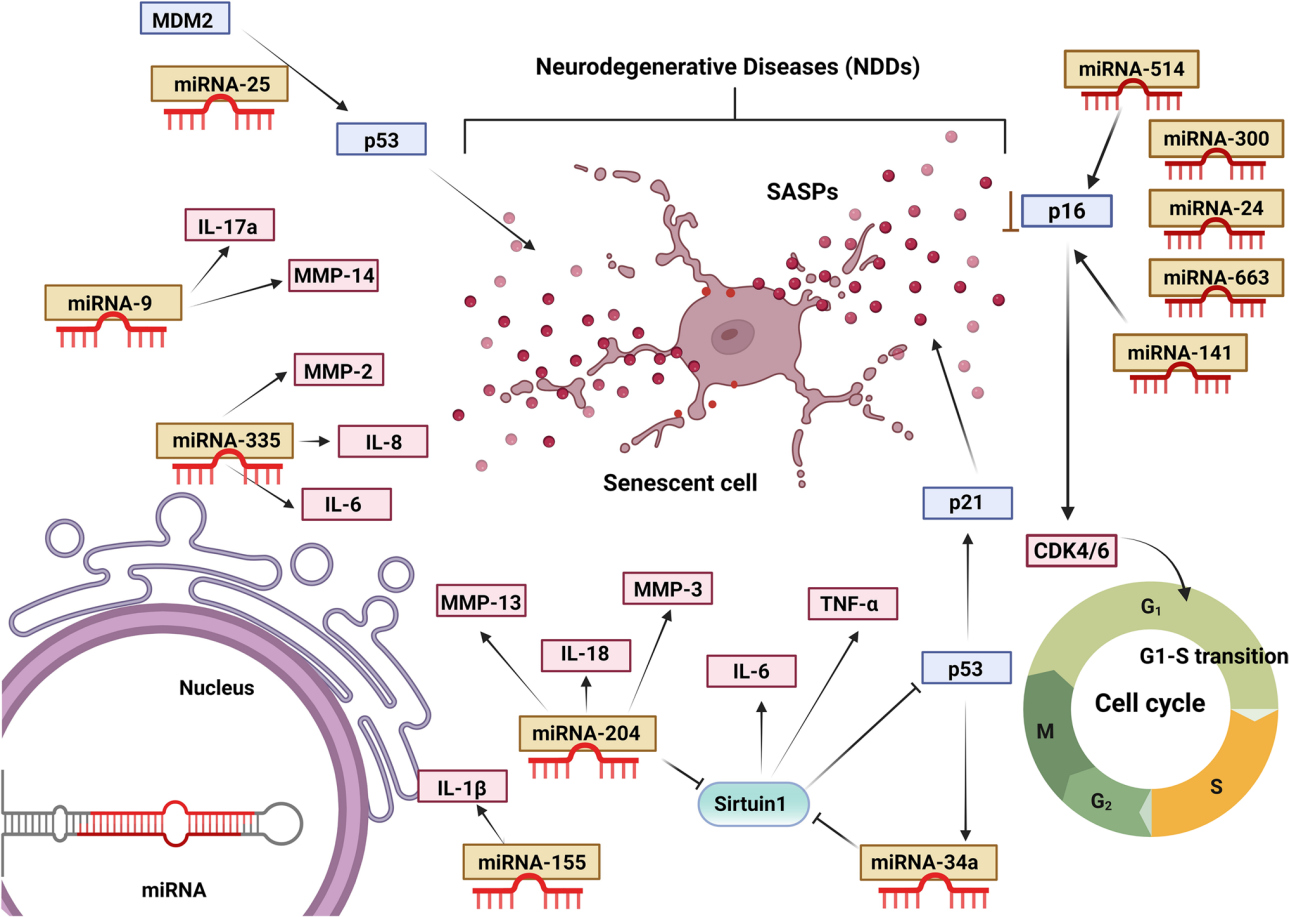
miRNAs and their associations with senescence. Various miRNAs, such as miRNA-25, miRNA-9, miRNA-335, miRNA-204, miRNA-155, and miRNA-34a, are known to modulate the levels of key cytokines and chemokines, also known as senescence-associated secretory phenotypes (SASPs). miRNAs are also involved in regulating the expression of p16, a CDK inhibitor. miRNAs such as miR-24, miR-300, miR-514, miR-663, and miR-141 can regulate the expression of p16. miR-24 directly binds to p16 to downregulate its translation, whereas the presence of miR-300, miR-514, miR-663 and miR-141 leads to p16 expression suppression

miRNA-335 and miRNA-9 are known to influence the function of matrix metalloproteinases (MMPs), such as MMP-14 and MMP-2. MMP-14 is a membrane-bound protein and activator of proMMP-2 that is present on glial cells and neurons. This activation leads to the degradation of laminin and collagen, which are ECM components, and results in leakage from the BBB, neuroinflammation, and neuronal death. The increased levels of these MMPs are also responsible for the progression of various neurodegenerative disorders, such as AD and MS. Hence, miRNAs such as miRNA-335 and miRNA-9 work by inhibiting these MMPs via the NF-kβ and SOX4 pathways, thus leading to reduced ECM degradation and an intact BBB (Fig. 4).

## Strategies to Target Senescent Cells in the Brain

Given the crucial role SnCs play in the development of NDDs and the involvement of SASPs in disease progression through the transfer of senescent phenotypes to nearby healthy cells, strategies to clear SnCs or hinder the production of SASPs have become critical therapeutic interventions. Senotherapeutics are a class of drugs that are both synthetic and natural and encompass two broad categories: senomorphics and senolytics. The primary objective of this class of drugs is to either selectively eliminate or delay the harmful repercussions of cellular senescence and, as a result, aging and age-associated diseases.

Senomorphics works by targeting the release and signaling of SASP molecules such as chemokines and cytokines. They are involved in blocking the transmission of senescence characteristics to nearby healthy cells, but do not cause the death of SnCs that secrete SASPs [88]. These senomorphics work primarily by targeting pathways such as the mTOR, p38MAPK, JAK/STAT, and PI3k/Akt pathways and transcription factors, including NF-κΒ, C/EBP β, and STAT3 [89]. Another approach adopted by senomorphics to target SASPs, such as IL-8 and IL-6, involves targeting them with associated antibodies. IL-8 is an important SASP that is overexpressed in senescent cells. Hence, there is an antibody, ABX-IL-8, that is designed to target the IL-8-secreting pathway, thus preventing its release [90]. Similarly, Mab-IL-6.8 is an antibody developed against the IL-6 SASP that prevents the secretion of this cytokine [91].

**Fig. 5:**
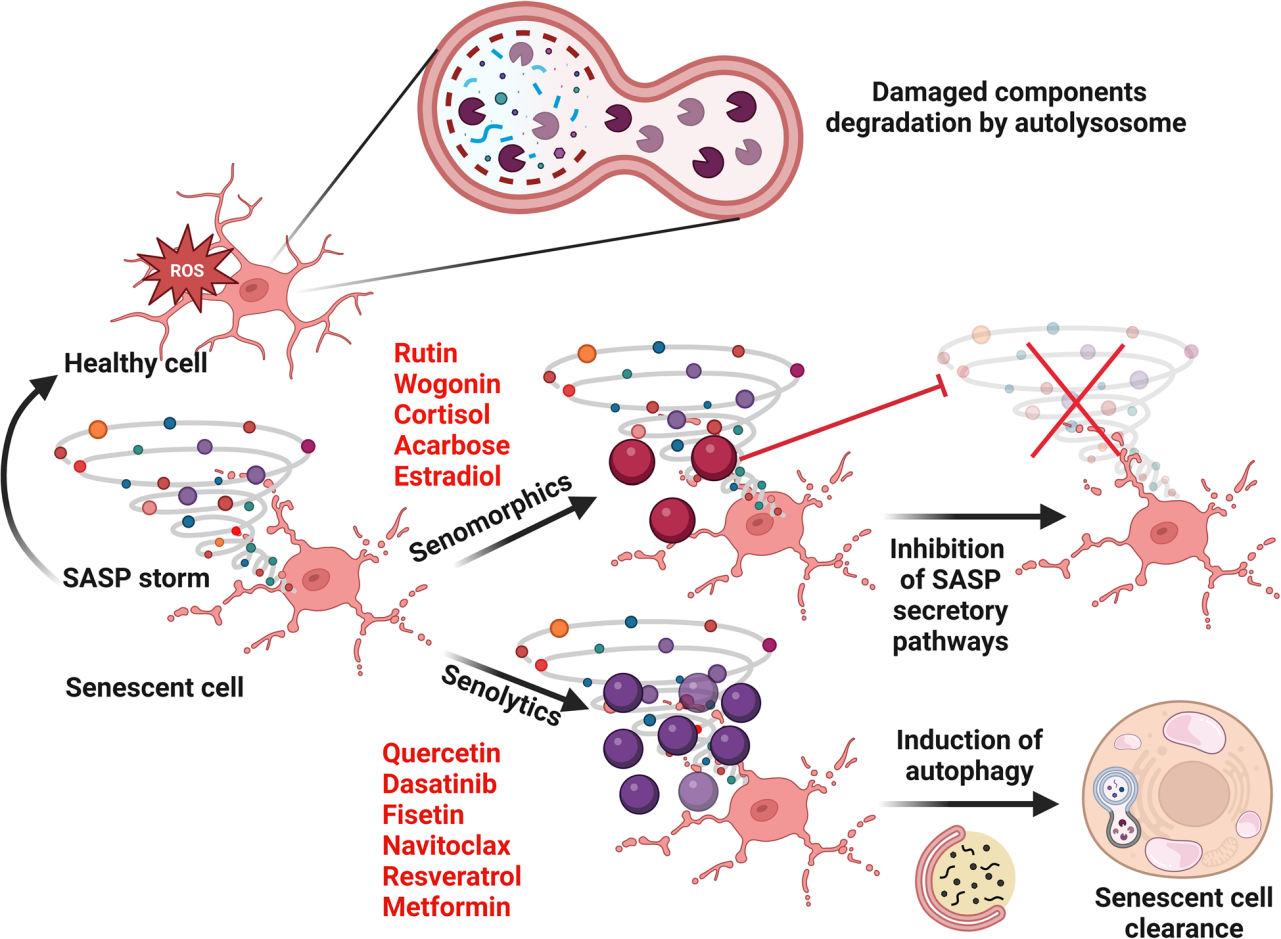
Senotherapeutics: senomorphics and senolytics. Senescent cells transmit senescent phenotypes to neighboring healthy cells via the secretion of SASPs. These senescent cells can be targeted via senotherapeutics such as senomorphics and senolytics. Senomorphics inhibit the production of SASPs from senescent cells, whereas senolytics specifically target and kill senescent cells via the induction of autophagy

The second category of senotherapeutics is senolytics, which, unlike senomorphics, target the death of senescent cells. The action of senolytics can interrupt, protect, or even reverse senescence, and promote health span and lifespan (Fig. 5).

## Senolytics

Senolytics are molecules that target and specifically cause the death of aged cells expressing the SASP. The use of senolytics with SnCs can be adopted as one of the approaches to treat age-associated NDDs. The main challenge in clearing SnCs is the ability to resist apoptotic stimuli, thus making most cytotoxic drugs available on the market inefficient. On the basis of this evidence, the principle of senolytic therapy is to selectively target proteins associated with senescent cell antiapoptotic pathways (SCAPs) by inhibiting or activating them to regulate the resistance of SnCs to apoptosis [92].

The first compound to be identified as a senolytic agent was through a drug discovery method. Because 30 to 70% of SnCs that have proapoptotic, tissue-damaging SASP phenotypes are unsusceptible to apoptosis, these aged cells rely on antiapoptotic, pro-survival pathways to avoid self-destruction [93]. Bioinformatics analysis revealed that one or more SCAPs are upregulated in SnCs. Transiently disabling SCAP pathways leads to the apoptosis of aged cells with a tissue-destructive SASP, whereas younger cells or senescent cells with a pro-growth, non-apoptotic SASP survive. The advantage of senolytics over SASP inhibitors is that SASP inhibitors potentially suppress both growth-promoting, proapoptotic, and inflammatory sets of SASP molecules. First-generation senolytics focus on the fundamental reason for detrimental SASP molecule generation by eliminating senescent cells that secrete proapoptotic agents. Quercetin and dasatinib were the first senolytics described in 2015 [93]. The names of several other well-studied and prominent senolytics, their sources, and their mechanisms of action are highlighted in Table 1.

**Table 1:** List of various senolytics and their sources and mechanisms of action

S. No	Senolytic agent	Source	Mechanism of action	References
1	Dasatinib	Synthetic drug, tyrosine kinase inhibitor	Inhibition of ephrins (EFNB-1/3)	[93, 95, 96]
2	Quercetin	Plant flavanol derived from onions, apples, grapes, berries, cherries, and citrus fruits	Inhibition of phosphatidylinositol 3-kinases (PI3K)	[95, 97, 98]
3	Fisetin	Plant flavonoid derived from strawberries, lotus root, and onions	Targets various pathways such as BCL-2, PI3K/AKT, p53, and NF-κB	[99–101]
4	Resveratrol	Plant polyphenols derived from grapes, berries, apples, and peanuts	Activation of SIRT1-mediated STAT3 signaling	[102–105]
5	Navitoclax (ABT-263)	Laboratory-developed synthetic drug	BCL2 family inhibitor	[106–108]
6	Piperlongumine (piplartine)	Amide alkaloid derived from long pepper (*Piper longum*)	Induces SnCs death by binding to oxidation resistance 1 (OXR1), leading to its proteasomal degradation	[109]
7	Curcumin	Lipophilic polyphenol derived from turmeric (*Curcuma longa*)	Downregulating the Nrf2 and NF-κB pathways	[110–113]
8	Rapamycin (sirolimus)	A macrolide derived from *Streptomyces hygroscopicus*, a bacterium present in soil	Inhibition of mTORC1	[114–117]
9	Metformin	*Galega officinalis *(French lilac)	Suppresses SASPs by inhibiting the phosphorylation of IκB and IKKα/β to block the translocation of NF-κB to the nucleus	[118–121]
10	Geldanamycin	Macrocyclic polyketide derived from *Streptomyces hygroscopicus*, a soil bacterium	HSP-90 inhibitor	[122]
11	Ouabain	Derived from seeds and barks of plants like *Acokanthera schimperi* and *Strophanthus gratus*	Inhibits the Na^+^/K^+^-ATPase, leading to destabilization of the membrane potential of senescent cells and intracellular acidification	[123–125]
12	Digoxin (brand name Lanoxin)	Derived from *Digitalis lanata *(Woolly foxglove)	Inhibits the Na^+^/K^+^-ATPase, leading to destabilization of the membrane potential of senescent cells and intracellular acidification	[123, 126]
13	Panobinostat	Synthetic histone deacetylase inhibitor (nonselective)	Histone deacetylase inhibitor, decreased expression of BCL-X_L_, and increased acetylated H3	[127, 128]
14	Alvespimycin (17-DMAG)	Synthetic derivative of natural senolytic geldanamycin	HSP-90 inhibitor disrupts the HSP90-AKT interaction to destabilize the active form of AKT	[122, 130, 130, 130]
15	EF24 curcumin analog	Synthetic analog of curcumin designed for increased bioavailability	Proteasome degradation of the Bcl-2 anti-apoptotic protein family proteins	[130]
16	Luteolin	Plant flavonoid derived from mint, broccoli, artichoke, celery, and oranges	Regulates NF-κΒ, JAK/STAT, and toll-like receptors (TLRs)	[130, 130, 130, 130]
17	Ruxolitinib	Synthetic drug	JAK1/2 inhibitor	[124, 130, 130]
18	FOXO-DRI	Synthetic peptide	Avoid the interaction between FOXO4 and p53	[130]
19	MitoTam	Synthetic drug	Decreases mitochondrial membrane potential and inhibits mitochondrial oxidative phosphorylation (OXPHOS), consequently exacerbating mitochondrial integrity	[130, 130, 130]
20	Tanespimycin (17-AAG)	Synthetic derivative of geldanamycin	HSP-90 inhibitor	[122, 130]

## Preclinical and Clinical Trial Reports

A study explored the neuroprotective effect of luteolin on brain aging via the administration of d-galactose. Male Wistar rats (*n* = 40; 2 months old, 160–190 g) were randomly categorized into four groups: the control group, the luteolin (80 mg/kg/day, i.p. in 1% DMSO for 1 month) group, the aging group (d-gal-induced) (150 mg/kg/day, 2.5 months), and the luteolin-treated aging group. All the abovementioned groups were subjected to behavioral analysis and assessment of cholinergic function and hippocampal mitochondrial respiration. In addition, oxidative stress in the hippocampus, senescence, neuroinflammation, and apoptotic marker expression have been observed. The expression levels of genes such as SIRT1, BDNF, and RAGE were analyzed. Histopathological analysis of the hippocampus, along with GFAP and Ki67 immunoreactivity, was also performed in this study. These results highlighted that luteolin is effective against d-gal-associated cognitive decline, resulting in the alleviation and reversal of cholinergic abnormalities. Furthermore, they concluded that the administration of luteolin alleviated oxidative stress in the hippocampus and mitigated the mitochondrial dysfunction, neuroinflammation, and senescence initiated by d-gal. Moreover, treatment with luteolin considerably decreased apoptosis in neurons and elevated the levels of SIRT1 mRNA expression in the hippocampus. This study revealed that the potential benefits of luteolin administration against d-gal-induced aging are associated with elevated expression of SIRT1 in the hippocampus, with subsequent modulation of the GLO1/AGE/RAGE signaling pathway [130].

Another study involving 0.5-year-old C57BL/6 J mice revealed that astrocytes and microglia present in the cerebral cortex, hippocampus, etc., colocalized with markers associated with cellular senescence, such as p16^Ink4a^ or p21^Cip1/Waf1^, at 5 weeks postinjury and 4 months postinjury in a model of controlled cortical impact. They reported that after senolytic dasatinib and quercetin (*D* + *Q*) administration intermittently started 1 month after traumatic brain injury (TBI) for 13 weeks, p16^Ink4a^-positive and p21^Cip1/Waf1^- + ve cells in the brains of TBI mice were prominently decreased, and the expression levels of major SASP-related proinflammatory markers, such as interleukin-1β and interleukin-6, were significantly reduced. They also reported that senolytic treatment decreased neurodegeneration and significantly increased the number of neurons present in the hippocampus and other prominent brain regions 18 weeks after TBI. Behavioral analysis after TBI (18 weeks) further revealed that senolytic treatment reversed impairments in memory, such as recognition and spatial reference memory, and alleviated depressive behavior in rodents with TBI [130].

However, another study explored the protective effects of quercetin against rotenone-induced Parkinson’s disease via the inhibition of NLRP3 inflammasome activation in microglia and the alleviation of mitochondrial dysregulation in male C57BL/6 J mice (2 months old). They hypothesized that curcumin could serve as a neuroprotective agent and a potential therapeutic for the treatment of PD. The animals were given curcumin at a concentration of 50 mg/kg per day or 0.1% DMSO IP for 21 days. They reported that treatment with curcumin significantly alleviated rotenone-induced behavioral abnormalities in mice, which was confirmed via the open field test, in which the total distance traveled was increased, and the rotarod test, in which the performance time was increased. Moreover, they reported that treatment with curcumin reversed the reduction in the number of dopaminergic neurons caused by rotenone administration in mice. Research has revealed that curcumin has neuroprotective effects on PD, including the inhibition of inflammation and oxidative stress, the reduction of monoamine oxidase B, and the inhibition of alpha-synuclein accumulation. Furthermore, curcumin inhibited NLRP3 inflammasome activation, cytokine overexpression, and NF-κB signaling pathway activation and increased mitochondrial fission in the SN in rotenone-treated mice. This study revealed that the protective effects of curcumin in PD are mediated through the NLRP3 inflammasome in microglia. Moreover, preclinical and clinical trial studies on various senolytics in neurodegenerative diseases are highlighted in Table 2.

**Table 2:** Preclinical and clinical trials of various senolytics for the treatment of neurodegenerative diseases

S. No	Neuro-degenerative disorder	Senolytics involved in treatment	Human/Animal model used	Dosage	Treatment duration	Inference	References
1	AD	Dasatinib + Quercetin	Human (mean age = 76 + 5 years; 40% female) and transgenic mouse model	Oral, 5 mg kg^−1^ D + 50 mg kg^−1^ Q (in transgenic mouse model) 100 mg D + 1000 mg Q (in humans)	Six repeating 2-week cycles of dose administration were conducted. Each cycle began with two consecutive days of study drug, followed by a 13- to 15-day study drug holiday	NCT04063124: Phase I clinical trial: CSF levels of interleukin-6 (IL-6) and glial fibrillary acidic protein (GFAP) increased (*t*(4) = 3.913, *P* = 0.008 and *t*(4) = 3.354, *P* = 0.028, respectively) with trending decreases in senescence-related cytokines and chemokines, and a trend toward higher Aβ42 levels (*t*(4) = − 2.338, *P* = 0.079) CNS penetrance of D, although Q was not detectable in CSF	SToMP-AD: NCT04063124 (Phase 1/2) Status: Completed 2023 Registration Date: 21 st August 2019 SToMP-AD RCT: NCT04685590 (Phase 2) Status: ongoing (expected 2027) Registration Date: 2021 [94] ALSENLITE: NCT04785300 (Phase 1/2) Status: Ongoing Registration Date: 4th March 2021 STAMINA (MCI + Slow Gait): NCT05422885 (Phase 1/2) Status: Ongoing Registration Date: 21 st June 2022
2	Cognitive Impairment	Metformin	Human (60–80 years old) total participants: 242	500–2000 mg nocte	Duration of the study: 3 years	NCT04511416: No results are public yet	MetMemory: NCT04511416 (Phase 3) Status: Ongoing Registration Date: 13th August 2020
3	Amnestic MCI or Mild AD dementia	Metformin	Human total participants: 20	Metformin 2000 mg/day	Duration of the study: 24 months	NCT05109169: Metformin was found to enhance memory and retention and was detected in CSF. No Significant change was observed in cerebral blood flow	METFINGER: NCT05109169 Status: Ongoing Registration Date: 2023
4	Mild Cognitive Impairment and Alzheimer’s Disease	Rapamycin	Human (55–85 years of age) total participants: 10 (60% female)	1 mg/day, orally	The duration of the study was 8 weeks	No changes in CSF were observed, indicating that rapamycin was not able to actively cross the blood–brain barrier at detectable levels Biomarkers related to AD pathology were inconclusive	RAPA Pilot: NCT04200911 (Early Phase 1) Status: Completed Results Posted Registration Date: 6th December 2019
5	Alzheimer’s and Cognitive Health	Rapamycin	Human total participants: 40	1 mg/day, orally	The drug was administered for 12 months (+ 3 days)	Results are not published yet Primary outcomes: Safety and tolerability testing of Rapamycin Secondary outcomes: Evaluate for CNS permeation of the drug and improvement in cognition	REACH: NCT04629495 9Phase 2) Status: Ongoing Registration Date: 11th September 2020
6	Alzheimer’s Disease	Rapamycin (Sirolimus)	Human (50–80 years of age) total participants: 15	7 mg/week, orally	The duration of the study is 6 months	Study ongoing, no results published yet Primary outcomes: Evaluate cerebral glucose metabolism Secondary outcomes: effect of the drug on age-related tissue changes	ERAP (PET Biomarker): NCT06022068 (Phase 1/2) Status: Ongoing Registration Date: 1 st September 2023
7	PD	Curcumin	Male C57BL/6 J mice (8 weeks old)	50 mg/kg/day, intraperitoneally	The drug was administered daily for 21 days	Inhibits inflammation and oxidative stress, reducing monoamine oxidase B, and inhibiting the aggregation of alpha-synuclein	[130]
8	Epilepsy and cognitive impairment induced by seizures	Metformin	Male C57BL/6 mice (4–6 weeks old)	200 mg/kg, intraperitoneally	Drug administration was performed on alternating days for 14 days	Metformin suppressed the progression of kindling, ameliorated the cognitive impairment, and decreased brain oxidative stress. It significantly decreased MDA level and increased GSH level relative to PTZ group. Therefore, it may be concluded that the antiepileptic effect of metformin is at least partly mediated through its antioxidant properties	[130]
9	PD	Resveratrol	Male A53T α-synuclein	Two dosages of resveratrol were compared: (1) 50 mg/kg body weight and (2) 10 mg/kg body weight	The drug was administered daily for 5 weeks	Protected against motor and cognitive deficits in A53T α-synuclein mice, lowered α-synuclein aggregate and oligomer levels, and reduced neuroinflammation and oxidative stress in the brains of A53T α-synuclein mice	[130]
10	MS	Navitoclax (ABT-263)	6–8-week-old C57Bl6 mice	1.5 mg/kg, intraperitoneally	The drug was administered daily for 18 days	Decreases disease severity, improves outcomes related to visual acuity and neuronal survival, and reduces white matter demyelination and inflammation	[130]
11	Spinal and bulbar muscular atrophy	17-DMAG	5-week-old male AR-24Q and AR-97Q mice	0.2 ml oral administration of 1 or 10 mg/kg (3 times a week, every other day)	11 weeks (starting from 5 to 16 weeks and 27 weeks for toxicity studies)	Ameliorated motor impairments in SBMA mice without detectable toxicity and reduced amounts of monomeric and nuclear-accumulated mutant androgen receptor (AR). Mutant AR was preferentially degraded in the presence of 17-DMAG in both SBMA cell and mouse models compared with wild-type AR. 17-DMAG also significantly induced Hsp70 and Hsp40	[130]

Given that the pathological hallmarks associated with senescence are found in ALS and that senescence leads to the progression of symptoms associated with ALS, such as elevated secretion of SASPs and decreased cellular functions, clearing senescent cells through the induction of autophagy can prove to be a useful strategy for alleviating ALS pathologies. Although there is a lack of research dealing with such therapeutic interventions to target ALS, the success of similar strategies in other NDs has focused on the fact that this approach can also be used for ALS [130].

## Nanocarrier-Based Senotherapeutics Delivery

Although there have been recent developments in targeting senescent cells in the brain, the present senotherapeutics face challenges in terms of bioavailability, nonspecific delivery, and metabolism in the gut, leading to ineffective therapeutic potential. This has led to the exploration of nanocarriers to deliver senotherapeutics efficiently to the brain. The health benefits of flavonoids, such as quercetin, resveratrol, and fisetin, have already been highlighted in this review; however, highlighting their poor pharmacokinetics when they are administered in their free form is also essential.

Various nanoformulations, such as liposomes, lipids, and polymeric nanoparticles, have been developed to address these issues. One study demonstrated the use of pegylated liposomes encapsulating rapamycin to study its anti-aging effect on aged human dermal fibroblasts induced by adriamycin. Compared with non-encapsulated rapamycin, they observed increased cell proliferation and migration as well as reduced expression of the senescence-associated markers SA-β-gal, p53, and p21 when compared to non-encapsulated rapamycin. Another study used poly lactic acid-glycolic acid (PLGA) nanoparticles to determine the effect of ABT263 on senescent cell clearance in a rat model. They concluded that nanoencapsulation enhanced the inhibition of inflammatory markers such as IL-6, the p16 senescence marker, and SASPs [130, 130].

In another study, navitoclax (2.5 mg/kg) encapsulated in mesoporous silica nanoparticles was administered in a palbociclib-induced aged mouse model. They reported that nanoencapsulation enhanced the apoptosis of senescent cells [130]. Mesoporous silica nanoparticles were also used for the anti-aging effect of RSV in the ApoE^−/−^ mice model in vivo. They concluded that nanoencapsulation facilitated improved inhibition of ROS generation, oxidation of low-density lipoprotein, and production of proinflammatory cytokines (IL-6 and TNF-α) [130]. While these nanoformulations have shown significant results in pre-clinical evaluation and in vitro models, accelerated clinical trials are needed to validate their effectiveness as a therapeutic option, as well as to check their safety in humans.

## Challenges and Future Perspective

Although the results from several animal models in which various senolytics have been administered have demonstrated their potential to improve brain-associated pathologies, their translation for use in clinical trials and eventually as therapeutic products still presents certain challenges. The nature of cellular senescence and the SASP is highly heterogeneous [130]. Although these senotherapeutics molecules have shown remarkable potential in the treatment of brain aging and associated NDDs, their variability in effectiveness across different brain regions and cell types is still a concern. For example, studies have shown that senescence in primary cortical astrocytes is associated with increased mTOR activation, autophagy suppression, and increased SASP production. Furthermore, treatment with the mTOR inhibitor rapamycin led to the suppression of SASP marker expression and restored homeostasis in cultured astrocytes. However, when microglia were subjected to the same intervention, variability in efficacy was observed compared with that in astrocytes. These findings suggest that different cell types can have different levels of reliance on senescence-associated pathways, which affects how different senotherapeutics act on them [130]. Apart from this, there is a difference in the activity of SIRT1 observed in the hippocampus and prefrontal cortex of the brain, which can lead to variation in the action of senotherapeutics such as resveratrol, which activate SIRT1. Therefore, it is essential to optimize senolytics for regional and cell-specific approaches to combat NDDs.

There is no single marker for characterizing either cellular senescence or SASPs, which makes assessing these diverse markers to identify phenotypes associated with senescence crucial and time-consuming. Therefore, when a senolytic agent is administered to treat disorders of the CNS, it is vital to understand the pathways that are targeted by specific senolytic molecules to understand the cascade of the response it elicits.

Another major hindrance associated with senolytics is that the survival pathways that they target are not necessarily exclusive to senescent cells. One prominent example of this is navitoclax. Bcl-XL inhibition at the desired site by navitoclax leads to the killing of platelets at other sites [130], with dose-limiting toxicity, primarily thrombocytopenia [130]. Absolute selectivity seems to be achievable only if a pro-survival mechanism that is utilized only by aged cells exists. However, this phenomenon has not yet been observed in vitro, as senolytics invariably cause the death of non-senescent healthy cells at excessive doses.

The brain is surrounded by the blood–brain barrier, which limits the entry of therapeutic agents. The development of senolytic compounds that can effectively cross the BBB at adequate concentrations without causing toxicity or damaging this protective barrier is a significant hurdle. Modifying drug properties or developing advanced delivery systems (e.g., nanoparticles) may be needed to ensure that senolytic therapies can reach target cells in the brain.

Several compounds with senolytic properties, such as dasatinib, quercetin, and navitoclax, are already FDA-approved for other conditions. Repurposing these drugs for treating neurodegenerative diseases and brain aging could accelerate the clinical application of senolytic therapies. While preclinical data have shown favorable results, large-scale clinical studies in humans are still in their infancy. Given the complexity of neurodegenerative conditions, patient selection, biomarkers, and outcome measures will require careful consideration in future trials.

## Conclusion

With age, there is an aggregation of senescent cells in various human and mouse tissues, and their removal was recently shown to be a potential therapeutic for the prevention of certain age-related pathologies. Neurodegeneration associated with these conditions is closely associated with neuroinflammation. A potent source of neuroinflammation is pro-inflammatory SASP from aged cells in the brain.

This review highlights that cellular senescence is a prominent pathological characteristic of NDDs, such as AD, PD, MS, and ALS. These disorders also tend to involve common signaling pathways that facilitate the promotion of senescence and facilitate this state of permanent cell arrest to initiate or worsen the symptoms of NDDs. Thus, the phenomenon of senescence needs to be investigated further, given its common links to pathways associated with neurodegeneration.

In addition, cellular senescence leads to an alteration in proteostasis in certain diseases, causing the formation of misfolded proteins. DNA damage, telomere dysfunction, oxidative stress, and neuroinflammation all contribute to the pathogenesis of NDDs. Pathologically induced and age-associated senescence are possible starts of cellular senescence in the brains of people with AD and other NDDs. Thus, eliminating senescent cells can influence outcomes.

Currently, there are no efficient neurotherapeutic options that can avert or retard the progression of neurogenerative disorders. Thus, novel neuroprotective therapeutic approaches are needed. In response to these drawbacks, different approaches, e.g., senolytics and senomorphics, are under investigation.

Senolytic treatments, which selectively target and eliminate senescent cells, offer promising therapeutic strategies for alleviating neurodegenerative diseases (NDDs) and mitigating brain aging. Cellular senescence, a state in which cells permanently cease dividing and acquire a proinflammatory secretory phenotype, plays a significant role in age-related tissue dysfunction, including in the brain. The accumulation of senescent cells in the central nervous system (CNS) has been implicated in promoting chronic inflammation, disrupting neural function, and contributing to the pathogenesis of various neurodegenerative disorders, such as Alzheimer’s disease and Parkinson’s disease.

Senescent cells, through SASP, can propagate local inflammation and neuronal damage, thereby accelerating brain aging. This chronic inflammatory environment compromises neuronal function, affects blood‒brain barrier integrity, and disrupts the balance of neural stem cell renewal, all of which are critical for maintaining cognitive function.

By employing senolytic therapies to selectively clear senescent cells from the brain, it is possible to reduce the inflammatory burden (SASP), restore tissue homeostasis, and preserve neuronal function. Preclinical studies have shown that senolytics can enhance cognitive function and neural plasticity and decrease neuroinflammation in aged animals, thereby demonstrating the potential to slow or even reverse aspects of brain aging and neurodegeneration.

However, there are certain concerns associated with the current senotherapeutics that need to be addressed to make the transition from preclinical to clinical trials smoother. Most senolytics face problems with BBB permeation, which restricts their bioavailability in the brain. One approach to mitigate this problem would be to increase the dosing or concentration of the senolytics, but that is again associated with issues such as systemic toxicity. Therefore, maintaining a safe dosage of senotherapeutics while ensuring bioavailability in the brain is difficult. Furthermore, senotherapeutics derived from natural compounds such as plant-based flavonoids are easily metabolized in the gut before they can penetrate the brain. Hence, effectively delivering these drugs to the brain is challenging. For pre-clinical studies, strategies such as stereotaxic or intranasal approaches are being used to combat this problem, but clinical studies need more non-invasive, effective, and easy-to-administer solutions. To address this issue, nano-delivery systems are being explored. Encapsulating senolytic compounds in nanocarriers can help increase their stability and facilitate the controlled release of these compounds. Furthermore, decorating the nanoparticle with the BBB and senescent cell-specific ligands can facilitate specific penetration into the brain and target only senescent cells, thus overcoming the issues of bioavailability and off-target effects. Presently, most clinical trials involving senotherapeutics are focused on systemic aging or cancer, whereas trials involving neurodegeneration and associated diseases such as AD are in the early stages and still require robust studies to establish their efficacy.

In conclusion, senolytic treatments provide a novel and targeted approach to combat neurodegenerative diseases and brain aging by directly addressing the detrimental impact of cellular senescence. The development of senolytics with greater selectivity and efficiency in targeting senescent cells while diminishing off-target effects on healthy cells is an ongoing challenge. While further research and clinical trials are necessary to assess their efficacy and safety in humans, these therapies hold the promise of significantly improving brain health in aging populations and individuals with neurodegenerative conditions.

## Data Availability

No datasets were generated or analysed during the current study.

## Declarations

### Declaration of Clinical Trials

This review article did not involve any new studies with human participants or animals performed by any of the authors. Hence, the clinical trial number is not applicable.

## References

[1] Fang Y, Peck MR, Quinn K, et al (2024) Senolytic intervention improves cognition, metabolism, and adiposity in female APP NL-F/NL-F mice. bioRxiv 2023.12.12.571277. 10.1101/2023.12.12.571277

[2] Alshaebi F, Sciortino A, Kayed R (2025) The role of glial cell senescence in Alzheimer’s disease. J Neurochem 169:e70051. 10.1111/jnc.70051 40130281 10.1111/jnc.70051 PMC11934031

[3] Di Micco R, Krizhanovsky V, Baker D, d’Adda di Fagagna F (2021) Cellular senescence in ageing: from mechanisms to therapeutic opportunities. Nat Rev Mol Cell Biol 22:75–95. 10.1038/s41580-020-00314-w 33328614 10.1038/s41580-020-00314-w PMC8344376

[4] Zhang L, Pitcher LE, Prahalad V et al (2023) Targeting cellular senescence with senotherapeutics: senolytics and senomorphics. FEBS J 290:1362–1383. 10.1111/febs.16350 35015337 10.1111/febs.16350

[5] Hayflick L, Moorhead PS (1961) The serial cultivation of human diploid cell strains. Exp Cell Res 25:585–621. 10.1016/0014-4827(61)90192-6 13905658 10.1016/0014-4827(61)90192-6

[6] Di Leonardo A, Linke SP, Clarkin K, Wahl GM (1994) DNA damage triggers a prolonged p53-dependent G1 arrest and long-term induction of Cip1 in normal human fibroblasts. Genes Dev 8:2540–2551. 10.1101/gad.8.21.2540 7958916 10.1101/gad.8.21.2540

[7] Kumari R, Jat P (2021) Mechanisms of cellular senescence: cell cycle arrest and senescence associated secretory phenotype. Front Cell Dev Biol. 10.3389/fcell.2021.645593 33855023 10.3389/fcell.2021.645593 PMC8039141

[8] Yoon Y-S, You JS, Kim T-K et al (2022) Senescence and impaired DNA damage responses in alpha-synucleinopathy models. Exp Mol Med 54:115–128. 10.1038/s12276-022-00727-x 35136202 10.1038/s12276-022-00727-x PMC8894476

[9] Miwa S, Kashyap S, Chini E, von Zglinicki T (2022) Mitochondrial dysfunction in cell senescence and aging. J Clin Invest 132:e158447. 10.1172/JCI158447 35775483 10.1172/JCI158447 PMC9246372

[10] Nousis L, Kanavaros P, Barbouti A (2023) Oxidative stress-induced cellular senescence: is labile iron the connecting link? Antioxidants 12:1250. 10.3390/antiox12061250 37371980 10.3390/antiox12061250 PMC10295026

[11] Rothkamm K, Krüger I, Thompson LH, Löbrich M (2003) Pathways of DNA double-strand break repair during the mammalian cell cycle. Mol Cell Biol 23:5706–5715. 10.1128/MCB.23.16.5706-5715.2003 12897142 10.1128/MCB.23.16.5706-5715.2003 PMC166351

[12] Rodier F, Campisi J, Bhaumik D (2007) Two faces of p53: aging and tumor suppression. Nucleic Acids Res 35:7475–7484. 10.1093/nar/gkm744 17942417 10.1093/nar/gkm744 PMC2190721

[13] Rossiello F, Jurk D, Passos JF, d’Adda di Fagagna F (2022) Telomere dysfunction in ageing and age-related diseases. Nat Cell Biol 24:135–147. 10.1038/s41556-022-00842-x 35165420 10.1038/s41556-022-00842-x PMC8985209

[14] Jeppesen DK, Bohr VA, Stevnsner T (2011) DNA repair deficiency in neurodegeneration. Prog Neurobiol 94:166–200. 10.1016/j.pneurobio.2011.04.013 21550379 10.1016/j.pneurobio.2011.04.013 PMC3123739

[15] Boengler K, Kosiol M, Mayr M et al (2017) Mitochondria and ageing: role in heart, skeletal muscle and adipose tissue. J Cachexia Sarcopenia Muscle 8:349–369. 10.1002/jcsm.12178 28432755 10.1002/jcsm.12178 PMC5476857

[16] Miwa S, Jow H, Baty K et al (2014) Low abundance of the matrix arm of complex I in mitochondria predicts longevity in mice. Nat Commun 5:3837. 10.1038/ncomms4837 24815183 10.1038/ncomms4837 PMC4024759

[17] Nacarelli T, Lau L, Fukumoto T et al (2019) NAD+ metabolism governs the proinflammatory senescence-associated secretome. Nat Cell Biol 21:397–407. 10.1038/s41556-019-0287-4 30778219 10.1038/s41556-019-0287-4 PMC6448588

[18] Tyagi A, Nguyen CU, Chong T et al (2018) Sirt3 deficiency-induced mitochondrial dysfunction and inflammasome formation in the brain. Sci Rep 8:17547. 10.1038/s41598-018-35890-7 30510203 10.1038/s41598-018-35890-7 PMC6277395

[19] Trifunovic A, Hansson A, Wredenberg A et al (2005) Somatic mtDNA mutations cause aging phenotypes without affecting reactive oxygen species production. Proc Natl Acad Sci U S A 102:17993–17998. 10.1073/pnas.0508886102 16332961 10.1073/pnas.0508886102 PMC1312403

[20] Wiley CD, Velarde MC, Lecot P et al (2016) Mitochondrial dysfunction induces senescence with a distinct secretory phenotype. Cell Metab 23:303–314. 10.1016/j.cmet.2015.11.011 26686024 10.1016/j.cmet.2015.11.011 PMC4749409

[21] López-Otín C, Blasco MA, Partridge L et al (2013) The hallmarks of aging. Cell 153:1194–1217. 10.1016/j.cell.2013.05.039 23746838 10.1016/j.cell.2013.05.039 PMC3836174

[22] Brunk UT, Terman A (2002) Lipofuscin: mechanisms of age-related accumulation and influence on cell function. Free Radic Biol Med 33:611–619. 10.1016/s0891-5849(02)00959-0 12208347 10.1016/s0891-5849(02)00959-0

[23] Bachstetter AD, Xing B, de Almeida L et al (2011) Microglial p38α MAPK is a key regulator of proinflammatory cytokine up-regulation induced by toll-like receptor (TLR) ligands or beta-amyloid (Aβ). J Neuroinflammation 8:79. 10.1186/1742-2094-8-79 21733175 10.1186/1742-2094-8-79 PMC3142505

[24] Coppé J-P, Desprez P-Y, Krtolica A, Campisi J (2010) The senescence-associated secretory phenotype: the dark side of tumor suppression. Annu Rev Pathol 5:99–118. 10.1146/annurev-pathol-121808-102144 20078217 10.1146/annurev-pathol-121808-102144 PMC4166495

[25] Moreno-Blas D, Gorostieta-Salas E, Pommer-Alba A et al (2019) Cortical neurons develop a senescence-like phenotype promoted by dysfunctional autophagy. Aging 11:6175–6198. 10.18632/aging.102181 31469660 10.18632/aging.102181 PMC6738425

[26] Campisi J, Kapahi P, Lithgow GJ et al (2019) From discoveries in ageing research to therapeutics for healthy ageing. Nature 571:183–192. 10.1038/s41586-019-1365-2 31292558 10.1038/s41586-019-1365-2 PMC7205183

[27] Herbig U, Ferreira M, Condel L et al (2006) Cellular senescence in aging primates. Science 311:1257. 10.1126/science.1122446 16456035 10.1126/science.1122446

[28] Yousefzadeh MJ, Wilkinson JE, Hughes B et al (2020) Heterochronic parabiosis regulates the extent of cellular senescence in multiple tissues. Geroscience 42:951–961. 10.1007/s11357-020-00185-1 32285290 10.1007/s11357-020-00185-1 PMC7286998

[29] Prattichizzo F, De Nigris V, La Sala L et al (2016) Inflammaging” as a druggable target: a senescence-associated secretory phenotype—centered view of type 2 diabetes. Oxid Med Cell Longev 2016:1810327. 10.1155/2016/1810327 27340505 10.1155/2016/1810327 PMC4908264

[30] Franceschi C, Garagnani P, Parini P et al (2018) Inflammaging: a new immune–metabolic viewpoint for age-related diseases. Nat Rev Endocrinol 14:576–590. 10.1038/s41574-018-0059-4 30046148 10.1038/s41574-018-0059-4

[31] Gallage S, Irvine EE, Barragan Avila JE et al (2024) Ribosomal S6 kinase 1 regulates inflammaging via the senescence secretome. Nat Aging. 10.1038/s43587-024-00695-z 39210150 10.1038/s43587-024-00695-z PMC11564105

[32] Peters R (2006) Ageing and the brain. Postgrad Med J 82:84–88. 10.1136/pgmj.2005.036665 16461469 10.1136/pgmj.2005.036665 PMC2596698

[33] Fjell AM, Westlye LT, Grydeland H et al (2014) Accelerating cortical thinning: unique to dementia or universal in aging? Cereb Cortex 24:919–934. 10.1093/cercor/bhs379 23236213 10.1093/cercor/bhs379 PMC3948495

[34] Bonifazi P, Erramuzpe A, Diez I et al (2018) Structure–function multi-scale connectomics reveals a major role of the fronto-striato-thalamic circuit in brain aging. Hum Brain Mapp 39:4663–4677. 10.1002/hbm.24312 30004604 10.1002/hbm.24312 PMC6866396

[35] Damoiseaux JS (2017) Effects of aging on functional and structural brain connectivity. Neuroimage 160:32–40. 10.1016/j.neuroimage.2017.01.077 28159687 10.1016/j.neuroimage.2017.01.077

[36] Salas IH, Burgado J, Allen NJ (2020) Glia: victims or villains of the aging brain? Neurobiol Dis 143:105008. 10.1016/j.nbd.2020.105008 32622920 10.1016/j.nbd.2020.105008

[37] Bitto A, Sell C, Crowe E et al (2010) Stress-induced senescence in human and rodent astrocytes. Exp Cell Res 316:2961–2968. 10.1016/j.yexcr.2010.06.021 20620137 10.1016/j.yexcr.2010.06.021

[38] Evans RJ, Wyllie FS, Wynford-Thomas D et al (2003) A P53-dependent, telomere-independent proliferative life span barrier in human astrocytes consistent with the molecular genetics of glioma development. Cancer Res 63:4854–4861 12941806

[39] Bhat R, Crowe EP, Bitto A et al (2012) Astrocyte senescence as a component of Alzheimer’s disease. PLoS One 7:e45069. 10.1371/journal.pone.0045069 22984612 10.1371/journal.pone.0045069 PMC3440417

[40] Wong WT (2013) Microglial aging in the healthy CNS: phenotypes, drivers, and rejuvenation. Front Cell Neurosci 7:22. 10.3389/fncel.2013.00022 23493481 10.3389/fncel.2013.00022 PMC3595516

[41] Ritzel RM, Doran SJ, Glaser EP et al (2019) Old age increases microglial senescence, exacerbates secondary neuroinflammation, and worsens neurological outcomes after acute traumatic brain injury in mice. Neurobiol Aging 77:194–206. 10.1016/j.neurobiolaging.2019.02.010 30904769 10.1016/j.neurobiolaging.2019.02.010 PMC6486858

[42] Tan FCC, Hutchison ER, Eitan E, Mattson MP (2014) Are there roles for brain cell senescence in aging and neurodegenerative disorders? Biogerontology 15:643–660. 10.1007/s10522-014-9532-1 25305051 10.1007/s10522-014-9532-1 PMC4264619

[43] Geng Y-Q, Guan J-T, Xu X-H, Fu Y-C (2010) Senescence-associated beta-galactosidase activity expression in aging hippocampal neurons. Biochem Biophys Res Commun 396:866–869. 10.1016/j.bbrc.2010.05.011 20457127 10.1016/j.bbrc.2010.05.011

[44] Dong W, Cheng S, Huang F, et al (2011) Mitochondrial dysfunction in long-term neuronal cultures mimics changes with aging. Med Sci Monit 17:BR91–96. 10.12659/msm.881706 10.12659/MSM.881706 PMC3539510 21455101

[45] Bhanu MU, Mandraju RK, Bhaskar C, Kondapi AK (2010) Cultured cerebellar granule neurons as an in vitro aging model: topoisomerase IIβ as an additional biomarker in DNA repair and aging. Toxicol In Vitro 24:1935–1945. 10.1016/j.tiv.2010.08.003 20708677 10.1016/j.tiv.2010.08.003

[46] Gunes S, Aizawa Y, Sugashi T et al (2022) Biomarkers for Alzheimer’s disease in the current state: a narrative review. Int J Mol Sci 23:4962. 10.3390/ijms23094962 35563350 10.3390/ijms23094962 PMC9102515

[47] Angelova DM, Brown DR (2019) Microglia and the aging brain: are senescent microglia the key to neurodegeneration? J Neurochem 151:676–688. 10.1111/jnc.14860 31478208 10.1111/jnc.14860

[48] Kirkland JL, Tchkonia T (2017) Cellular senescence: a translational perspective. EBioMedicine 21:21–28. 10.1016/j.ebiom.2017.04.013 28416161 10.1016/j.ebiom.2017.04.013 PMC5514381

[49] Flanary BE, Streit WJ (2004) Progressive telomere shortening occurs in cultured rat microglia, but not astrocytes. Glia 45:75–88. 10.1002/glia.10301 14648548 10.1002/glia.10301

[50] Zhu J, Wu C, Yang L (2024) Cellular senescence in Alzheimer’s disease: from physiology to pathology. Transl Neurodegener 13:55. 10.1186/s40035-024-00447-4 39568081 10.1186/s40035-024-00447-4 PMC11577763

[51] Dorigatti AO, Riordan R, Yu Z et al (2022) Brain cellular senescence in mouse models of Alzheimer’s disease. Geroscience 44:1157–1168. 10.1007/s11357-022-00531-5 35249206 10.1007/s11357-022-00531-5 PMC9135905

[52] Dorsey ER, Elbaz A, Nichols E et al (2018) Global, regional, and national burden of Parkinson’s disease, 1990–2016: a systematic analysis for the Global Burden of Disease Study 2016. Lancet Neurol 17:939–953. 10.1016/S1474-4422(18)30295-3 30287051 10.1016/S1474-4422(18)30295-3 PMC6191528

[53] Chinta SJ, Woods G, Demaria M et al (2018) Cellular senescence is induced by the environmental neurotoxin paraquat and contributes to neuropathology linked to Parkinson’s disease. Cell Rep 22:930–940. 10.1016/j.celrep.2017.12.092 29386135 10.1016/j.celrep.2017.12.092 PMC5806534

[54] van Duijn CM, Dekker MCJ, Bonifati V et al (2001) PARK7, a novel locus for autosomal recessive early-onset parkinsonism, on chromosome 1p36. Am J Hum Genet 69:629–634. 10.1086/322996 11462174 10.1086/322996 PMC1235491

[55] Nicaise AM, Wagstaff LJ, Willis CM et al (2019) Cellular senescence in progenitor cells contributes to diminished remyelination potential in progressive multiple sclerosis. Proc Natl Acad Sci U S A 116:9030–9039. 10.1073/pnas.1818348116 30910981 10.1073/pnas.1818348116 PMC6500153

[56] Yang JH, Miner AE, Fair A et al (2024) Senescence marker p16INK4a expression in patients with multiple sclerosis. Mult Scler Relat Disord 84:105498. 10.1016/j.msard.2024.105498 38359693 10.1016/j.msard.2024.105498

[57] Gross PS, Durán-Laforet V, Ho LT et al (2025) Senescent-like microglia limit remyelination through the senescence associated secretory phenotype. Nat Commun 16:2283. 10.1038/s41467-025-57632-w 40055369 10.1038/s41467-025-57632-w PMC11889183

[58] Brotman RG, Moreno-Escobar MC, Joseph J, et al (2024) Amyotrophic lateral sclerosis. In: StatPearls. StatPearls Publishing, Treasure Island (FL) 32310611

[59] Trias E, Beilby PR, Kovacs M et al (2019) Emergence of microglia bearing senescence markers during paralysis progression in a rat model of inherited ALS. Front Aging Neurosci 11:42. 10.3389/fnagi.2019.00042 30873018 10.3389/fnagi.2019.00042 PMC6403180

[60] Vazquez-Villaseñor I, Garwood CJ, Heath PR et al (2020) Expression of p16 and p21 in the frontal association cortex of ALS/MND brains suggests neuronal cell cycle dysregulation and astrocyte senescence in early stages of the disease. Neuropathol Appl Neurobiol 46:171–185. 10.1111/nan.12559 31077599 10.1111/nan.12559 PMC7217199

[61] Carling D (2017) AMPK signalling in health and disease. Curr Opin Cell Biol 45:31–37. 10.1016/j.ceb.2017.01.005 28232179 10.1016/j.ceb.2017.01.005

[62] Paudel YN, Angelopoulou E, Piperi C et al (2020) Emerging neuroprotective effect of metformin in Parkinson’s disease: a molecular crosstalk. Pharmacol Res 152:104593. 10.1016/j.phrs.2019.104593 31843673 10.1016/j.phrs.2019.104593

[63] Yu M, Zhang H, Wang B et al (2021) Key signaling pathways in aging and potential interventions for healthy aging. Cells 10:660. 10.3390/cells10030660 33809718 10.3390/cells10030660 PMC8002281

[64] Sharma A, Singh AK (2023) Molecular mechanism of caloric restriction mimetics-mediated neuroprotection of age-related neurodegenerative diseases: an emerging therapeutic approach. Biogerontology 24:679–708. 10.1007/s10522-023-10045-y 37428308 10.1007/s10522-023-10045-y

[65] Dello Russo C, Cappoli N, Coletta I et al (2018) The human microglial HMC3 cell line: where do we stand? A systematic literature review. J Neuroinflammation 15:259. 10.1186/s12974-018-1288-0 30200996 10.1186/s12974-018-1288-0 PMC6131758

[66] Halloran J, Hussong SA, Burbank R et al (2012) Chronic inhibition of mammalian target of rapamycin by rapamycin modulates cognitive and non-cognitive components of behavior throughout lifespan in mice. Neuroscience 223:102–113. 10.1016/j.neuroscience.2012.06.054 22750207 10.1016/j.neuroscience.2012.06.054 PMC3454865

[67] Salminen A, Ojala J, Kaarniranta K et al (2011) Astrocytes in the aging brain express characteristics of senescence-associated secretory phenotype. Eur J Neurosci 34:3–11. 10.1111/j.1460-9568.2011.07738.x 21649759 10.1111/j.1460-9568.2011.07738.x

[68] Wang J, Gallagher D, DeVito LM et al (2012) Metformin activates an atypical PKC-CBP pathway to promote neurogenesis and enhance spatial memory formation. Cell Stem Cell 11:23–35. 10.1016/j.stem.2012.03.016 22770240 10.1016/j.stem.2012.03.016

[69] Michán S, Li Y, Chou MM-H et al (2010) SIRT1 is essential for normal cognitive function and synaptic plasticity. J Neurosci 30:9695–9707. 10.1523/JNEUROSCI.0027-10.2010 20660252 10.1523/JNEUROSCI.0027-10.2010 PMC2921958

[70] Kaushik S, Cuervo AM (2015) Proteostasis and aging. Nat Med 21:1406–1415. 10.1038/nm.4001 26646497 10.1038/nm.4001

[71] Komastu M (2022) P62 bodies: phase separation, NRF2 activation, and selective autophagic degradation. IUBMB Life 74:1200–1280. 10.1002/iub.2689 36331376 10.1002/iub.2689

[72] Zhang J, Wang P, Wan L et al (2017) The emergence of noncoding RNAs as Heracles in autophagy. Autophagy 13:1004–1024. 10.1080/15548627.2017.1312041 28441084 10.1080/15548627.2017.1312041 PMC5486373

[73] Wang X, Zhang M, Liu H (2019) LncRNA17A regulates autophagy and apoptosis of SH-SY5Y cell line as an in vitro model for Alzheimer’s disease. Biosci Biotechnol Biochem 83:609–621. 10.1080/09168451.2018.1562874 30652945 10.1080/09168451.2018.1562874

[74] Huang Z, Zhao J, Wang W et al (2020) Depletion of LncRNA NEAT1 rescues mitochondrial dysfunction through NEDD4L-dependent PINK1 degradation in animal models of Alzheimer’s disease. Front Cell Neurosci 14:28. 10.3389/fncel.2020.00028 32140098 10.3389/fncel.2020.00028 PMC7043103

[75] Kawajiri S, Saiki S, Sato S et al (2010) PINK1 is recruited to mitochondria with parkin and associates with LC3 in mitophagy. FEBS Lett 584:1073–1079. 10.1016/j.febslet.2010.02.016 20153330 10.1016/j.febslet.2010.02.016

[76] Xu X, Cui L, Zhong W, Cai Y (2020) Autophagy-associated lncRNAs: promising targets for neurological disease diagnosis and therapy. Neural Plast 2020:8881687. 10.1155/2020/8881687 33029125 10.1155/2020/8881687 PMC7528122

[77] Nishimoto Y, Nakagawa S, Hirose T et al (2013) The long non-coding RNA nuclear-enriched abundant transcript 1_2 induces paraspeckle formation in the motor neuron during the early phase of amyotrophic lateral sclerosis. Mol Brain 6:31. 10.1186/1756-6606-6-31 23835137 10.1186/1756-6606-6-31 PMC3729541

[78] Zhou S, Yu X, Wang M et al (2021) Long non-coding RNAs in pathogenesis of neurodegenerative diseases. Front Cell Dev Biol 9:719247. 10.3389/fcell.2021.719247 34527672 10.3389/fcell.2021.719247 PMC8435612

[79] Ma X, Zheng Q, Zhao G et al (2020) Regulation of cellular senescence by microRNAs. Mech Ageing Dev 189:111264. 10.1016/j.mad.2020.111264 32450085 10.1016/j.mad.2020.111264

[80] Bu H, Wedel S, Cavinato M, Jansen-Dürr P (2017) MicroRNA regulation of oxidative stress-induced cellular senescence. Oxid Med Cell Longev 2017:2398696. 10.1155/2017/2398696 28593022 10.1155/2017/2398696 PMC5448073

[81] Li S, Lei Z, Sun T (2023) The role of microRNAs in neurodegenerative diseases: a review. Cell Biol Toxicol 39:53–83. 10.1007/s10565-022-09761-x 36125599 10.1007/s10565-022-09761-x PMC9486770

[82] Williams J, Smith F, Kumar S et al (2017) Are microRNAs true sensors of ageing and cellular senescence? Ageing Res Rev 35:350–363. 10.1016/j.arr.2016.11.008 27903442 10.1016/j.arr.2016.11.008 PMC5357446

[83] Fulzele S, Mendhe B, Khayrullin A et al (2019) Muscle-derived miR-34a increases with age in circulating extracellular vesicles and induces senescence of bone marrow stem cells. Aging Albany NY 11:1791. 10.18632/aging.101874 30910993 10.18632/aging.101874 PMC6461183

[84] Raucci A, Vinci MC (2020) MiR-34a: a promising target for inflammaging and age-related diseases. Int J Mol Sci 21:8293. 10.3390/ijms21218293 33167452 10.3390/ijms21218293 PMC7663903

[85] Zuccolo E, Badi I, Scavello F et al (2020) The microrna-34a-induced senescence-associated secretory phenotype (SASP) favors vascular smooth muscle cells calcification. Int J Mol Sci 21:4454. 10.3390/ijms21124454 32585876 10.3390/ijms21124454 PMC7352675

[86] Gaudet AD, Fonken LK, Watkins LR et al (2018) Micrornas: roles in regulating neuroinflammation. Neuroscientist 24:221–245. 10.1177/1073858417721150 28737113 10.1177/1073858417721150 PMC8377730

[87] Migdalska-Sęk M, Góralska K, Jabłoński S et al (2020) Evaluation of the relationship between the IL-17A gene expression level and regulatory miRNA-9 in relation to tumor progression in patients with non-small cell lung cancer: a pilot study. Mol Biol Rep 47:583–592. 10.1007/s11033-019-05164-0 31707599 10.1007/s11033-019-05164-0

[88] Zhang L-M, Zhang J, Zhang Y et al (2019) Interleukin-18 promotes fibroblast senescence in pulmonary fibrosis through down-regulating Klotho expression. Biomed Pharmacother 113:108756. 10.1016/j.biopha.2019.108756 30870716 10.1016/j.biopha.2019.108756

[89] Lagoumtzi SM, Chondrogianni N (2021) Senolytics and senomorphics: natural and synthetic therapeutics in the treatment of aging and chronic diseases. Free Radic Biol Med 171:169–190. 10.1016/j.freeradbiomed.2021.05.003 33989756 10.1016/j.freeradbiomed.2021.05.003

[90] Song S, Tchkonia T, Jiang J et al (2020) Targeting senescent cells for a healthier aging: challenges and opportunities. Adv Sci 7:2002611. 10.1002/advs.202002611 10.1002/advs.202002611 PMC7709980 33304768

[91] Yang XD, Corvalan JR, Wang P et al (1999) Fully human anti-interleukin-8 monoclonal antibodies: potential therapeutics for the treatment of inflammatory disease states. J Leukoc Biol 66:401–410. 10.1002/jlb.66.3.401 10496309 10.1002/jlb.66.3.401

[92] Kuilman T, Michaloglou C, Vredeveld LCW et al (2008) Oncogene-induced senescence relayed by an interleukin-dependent inflammatory network. Cell 133:1019–1031. 10.1016/j.cell.2008.03.039 18555778 10.1016/j.cell.2008.03.039

[93] Lelarge V, Capelle R, Oger F, et al (2024) Senolytics: from pharmacological inhibitors to immunotherapies, a promising future for patients’ treatment. npj Aging 10:1–8. 10.1038/s41514-024-00138-4 10.1038/s41514-024-00138-4 PMC10847408 38321020

[94] Zhu Y, Tchkonia T, Pirtskhalava T et al (2015) The Achilles’ heel of senescent cells: from transcriptome to senolytic drugs. Aging Cell 14:644–658. 10.1111/acel.12344 25754370 10.1111/acel.12344 PMC4531078

[95] Gonzales MM, Garbarino VR, Kautz TF et al (2023) Senolytic therapy in mild Alzheimer’s disease: a phase 1 feasibility trial. Nat Med 29:2481–2488. 10.1038/s41591-023-02543-w 37679434 10.1038/s41591-023-02543-w PMC10875739

[96] Gu C, Guo T, Chen X et al (2025) Senolytic treatment attenuates global ischemic brain injury and enhances cognitive recovery by targeting mitochondria. Cell Mol Neurobiol 45:60. 10.1007/s10571-025-01580-y 40533653 10.1007/s10571-025-01580-y PMC12177126

[97] Darvesh S, Cash MK, Forrestall K, et al (2025) Differential senolytic inhibition of normal versus Aβ-associated cholinesterases: implications in aging and Alzheimer’s disease. Aging (Albany NY) 17:822–850. 10.18632/aging.206227 10.18632/aging.206227 PMC11984419 40159237

[98] Rad AN, Grillari J (2024) Current senolytics: mode of action, efficacy and limitations, and their future. Mech Ageing Dev 217:111888. 10.1016/j.mad.2023.111888 38040344 10.1016/j.mad.2023.111888

[99] Manavi Z, Melchor GS, Bullard MR et al (2025) Senescent cell reduction does not improve recovery in mice under experimental autoimmune encephalomyelitis (EAE) induced demyelination. J Neuroinflammation 22:101. 10.1186/s12974-025-03425-3 40197319 10.1186/s12974-025-03425-3 PMC11974124

[100] Syed DN, Adhami VM, Khan N et al (2016) Exploring the molecular targets of dietary flavonoid fisetin in cancer. Semin Cancer Biol 40:130–140. 10.1016/j.semcancer.2016.04.003 27163728 10.1016/j.semcancer.2016.04.003 PMC5067175

[101] Della Vedova L, Baron G, Morazzoni P et al (2025) The potential of polyphenols in modulating the cellular senescence process: implications and mechanism of action. Pharmaceuticals 18:138. 10.3390/ph18020138 40005954 10.3390/ph18020138 PMC11858549

[102] Dehkordi AJ, Jazi AA, Dehkordi KJ (2025) The effect of fisetin supplementation and high-intensity interval training on neurogenesis markers in aged Alzheimer’s model mice. Journal of Nutrition, Fasting & Health 13:35–43. 10.22038/JNFH.2024.82094.1528

[103] Liu J, Jiao K, Zhou Q et al (2021) Resveratrol alleviates 27-hydroxycholesterol-induced senescence in nerve cells and affects zebrafish locomotor behavior via activation of SIRT1-mediated STAT3 signaling. Oxid Med Cell Longev 2021:6673343. 10.1155/2021/6673343 34239694 10.1155/2021/6673343 PMC8238615

[104] Quincozes-Santos A, Bobermin LD, Tramontina AC et al (2025) Glioprotective effects of resveratrol against glutamate-induced cellular dysfunction: the role of heme oxygenase 1 pathway. Neurotox Res 43:7. 10.1007/s12640-025-00730-w 39869271 10.1007/s12640-025-00730-w

[105] Zhang YF, Xu ZL, Wang C et al (2025) Resveratrol attenuates prenatal X-ray-induced microcephaly and adult depression via SIRT1-mediated senescence suppression and TPH2/5-HT pathway restoration in mice. Phytomedicine 143:156845. 10.1016/j.phymed.2025.156845 40440905 10.1016/j.phymed.2025.156845

[106] Centonze M, Aloisio Caruso E, De Nunzio V et al (2025) The antiaging potential of dietary plant-based polyphenols: a review on their role in cellular senescence modulation. Nutrients 17:1716. 10.3390/nu17101716 40431456 10.3390/nu17101716 PMC12114605

[107] Yosef R, Pilpel N, Tokarsky-Amiel R et al (2016) Directed elimination of senescent cells by inhibition of BCL-W and BCL-XL. Nat Commun 7:11190. 10.1038/ncomms11190 27048913 10.1038/ncomms11190 PMC4823827

[108] Hartati I, Paramita V, Maharani F et al (2025) A brief review on aging and its combination with bibliometric analysis on cellular senescence elimination as part of anti aging strategies. AIP Conf Proc 3250:030004. 10.1063/5.0240687

[109] Palabiyik AA (2025) The role of Bcl-2 in controlling the transition between autophagy and apoptosis (Review). Mol Med Rep 32:1–9. 10.3892/mmr.2025.13537 10.3892/mmr.2025.13537 PMC12045647 40242969

[110] Zhang X, Zhang S, Liu X et al (2018) Oxidation resistance 1 is a novel senolytic target. Aging Cell 17:e12780. 10.1111/acel.12780 29766639 10.1111/acel.12780 PMC6052462

[111] Cherif H, Bisson DG, Jarzem P et al (2019) Curcumin and o-vanillin exhibit evidence of senolytic activity in human IVD cells *in vitro*. J Clin Med 8:433. 10.3390/jcm8040433 30934902 10.3390/jcm8040433 PMC6518239

[112] He Y, Liu Y, Zhang M (2025) The beneficial effects of curcumin on aging and age-related diseases: from oxidative stress to antioxidant mechanisms, brain health and apoptosis. Front Aging Neurosci. 10.3389/fnagi.2025.1533963 39906716 10.3389/fnagi.2025.1533963 PMC11788355

[113] Moselhy OA, Abdel-Aziz N, El-bahkery A et al (2025) Curcumin nanoparticles alleviate brain mitochondrial dysfunction and cellular senescence in γ-irradiated rats. Sci Rep 15:3857. 10.1038/s41598-025-87635-y 39890961 10.1038/s41598-025-87635-y PMC11785741

[114] Singh A, Soni U, Varadwaj PK et al (2025) Anti-inflammatory effect of curcumin in an accelerated senescence model of Wistar rat: an in vivo and in-silico study. J Biomol Struct Dyn 43:1459–1470. 10.1080/07391102.2023.2291832 38088364 10.1080/07391102.2023.2291832

[115] Schreiber KH, Arriola Apelo SI, Yu D et al (2019) A novel rapamycin analog is highly selective for mTORC1 *in vivo*. Nat Commun 10:3194. 10.1038/s41467-019-11174-0 31324799 10.1038/s41467-019-11174-0 PMC6642166

[116] Ge Q, Yang J, Huang F et al (2025) Multimodal single-cell analyses reveal molecular markers of neuronal senescence in human drug-resistant epilepsy. J Clin Invest. 10.1172/JCI188942 40026248 10.1172/JCI188942 PMC11870744

[117] Gonzales MM, Garbarino VR, Kautz TF et al (2025) Rapamycin treatment for Alzheimer’s disease and related dementias: a pilot phase 1 clinical trial. Commun Med 5:189. 10.1038/s43856-025-00904-9 40394335 10.1038/s43856-025-00904-9 PMC12092812

[118] Smith P, Carroll B (2025) Senescence in the ageing skin: a new focus on mTORC1 and the lysosome. FEBS J 292:960–975. 10.1111/febs.17281 39325694 10.1111/febs.17281 PMC11880983

[119] Moiseeva O, Deschênes-Simard X, St-Germain E et al (2013) Metformin inhibits the senescence-associated secretory phenotype by interfering with IKK/NF-κB activation. Aging Cell 12:489–498. 10.1111/acel.12075 23521863 10.1111/acel.12075

[120] Demir EI, Sezer G (2025) Metformin prevents oxidative stress-induced premature senescence in astrocyte cells. Authorea. 10.22541/au.173875324.47590402/v1

[121] Cho SI, Jo E-R, Jang HS (2025) Metformin alleviates auditory cell senescence by mitophagy induction. Neurosci Res 213:86–94. 10.1016/j.neures.2025.02.008 40023376 10.1016/j.neures.2025.02.008

[122] Zhang T, Zhou L, Makarczyk MJ et al (2025) The anti-aging mechanism of metformin: from molecular insights to clinical applications. Molecules 30:816. 10.3390/molecules30040816 40005128 10.3390/molecules30040816 PMC11858480

[123] Fuhrmann-Stroissnigg H, Ling YY, Zhao J et al (2017) Identification of HSP90 inhibitors as a novel class of senolytics. Nat Commun 8:422. 10.1038/s41467-017-00314-z 28871086 10.1038/s41467-017-00314-z PMC5583353

[124] Guerrero A, Herranz N, Sun B et al (2019) Cardiac glycosides are broad-spectrum senolytics. Nat Metab 1:1074–1088. 10.1038/s42255-019-0122-z 31799499 10.1038/s42255-019-0122-z PMC6887543

[125] Rademacher DJ, Exline JE, Foecking EM (2025) Role of cellular senescence in Parkinson’s disease: potential for disease-modification through senotherapy. Biomedicines 13:1400. 10.3390/biomedicines13061400 40564120 10.3390/biomedicines13061400 PMC12190713

[126] Nadeem J, Sultana R, Parveen A, Kim SY (2025) Recent advances in anti-aging therapeutic strategies targeting DNA damage response and senescence-associated secretory phenotype-linked signaling cascade. Cell Biochem Funct 43:e70046. 10.1002/cbf.70046 40008426 10.1002/cbf.70046

[127] Martín-Vicente P, López-Martínez C, López-Alonso I et al (2025) Mechanical stretch induces senescence of lung epithelial cells and drives fibroblast activation by paracrine mechanisms. Am J Respir Cell Mol Biol 72:195–205. 10.1165/rcmb.2023-0449OC 39133930 10.1165/rcmb.2023-0449OC

[128] Samaraweera L, Adomako A, Rodriguez-Gabin A, McDaid HM (2017) A novel indication for panobinostat as a senolytic drug in NSCLC and HNSCC. Sci Rep 7:1900. 10.1038/s41598-017-01964-1 28507307 10.1038/s41598-017-01964-1 PMC5432488

[129] Jaros M, Melk A (2025) Senotherapy: implications for transplantation. Transplantation. 109(7):1138-1151. 10.1097/TP.0000000000005291 10.1097/TP.0000000000005291 39885624

[130] Ferreira-Gonzalez S, Matsumoto T, Hara E, Forbes SJ (2025) Senescence, aging and disease throughout the gastrointestinal system. Gastroenterology. 10.1053/j.gastro.2025.06.010 40532827 10.1053/j.gastro.2025.06.010

[131] Nunes ADC, Pitcher LE, Exner HA et al (2025) Attenuation of cellular senescence and improvement of osteogenic differentiation capacity of human liver stem cells using specific senomorphic and senolytic agents. Stem Cell Rev Rep. 10.1007/s12015-025-10876-x 40220121 10.1007/s12015-025-10876-x PMC12316771

[132] Wu J, Liu X, Liu Y et al (2025) New insights into the role of cellular senescence and its therapeutic implications in ocular diseases. Bioengineering 12:563. 10.3390/bioengineering12060563 40564380 10.3390/bioengineering12060563 PMC12189812

[133] Li W, He Y, Zhang R et al (2019) The curcumin analog EF24 is a novel senolytic agent. Aging (Albany NY) 11:771–782. 10.18632/aging.101787 30694217 10.18632/aging.101787 PMC6366974

[134] Gendrisch F, Esser PR, Schempp CM, Wölfle U (2021) Luteolin as a modulator of skin aging and inflammation. Biofactors 47:170–180. 10.1002/biof.1699 33368702 10.1002/biof.1699

[135] Yan Y, Huang H, Su T et al (2025) Luteolin mitigates photoaging caused by uva-induced fibroblast senescence by modulating oxidative stress pathways. Int J Mol Sci 26:1809. 10.3390/ijms26051809 40076436 10.3390/ijms26051809 PMC11899068

[136] Singh NK, Bhushan B, Singh P, Sahu KK (2025) Therapeutic expedition of luteolin against brain-related disorders: an updated review. Comb Chem High Throughput Screen 28:371–391. 10.2174/0113862073303342240409060918 38659259 10.2174/0113862073303342240409060918

[137] Luo C, Wang Z, Liao J et al (2025) Luteolin ameliorates kainic acid-induced seizure by modulating GADD45B and reducing oxidative stress in hippocampal neurons. Neuropeptides 111:102524. 10.1016/j.npep.2025.102524 40441030 10.1016/j.npep.2025.102524

[138] Xu M, Tchkonia T, Ding H et al (2015) JAK inhibition alleviates the cellular senescence-associated secretory phenotype and frailty in old age. Proc Natl Acad Sci U S A 112:E6301-6310. 10.1073/pnas.1515386112 26578790 10.1073/pnas.1515386112 PMC4655580

[139] Abdullah AN, Zamri AF, Alias K et al (2025) Transient prenatal ruxolitinib treatment promotes neurogenesis and suppresses astrogliogenesis during embryonic mouse brain development. Neurosci Res Notes 8:352.1-352.7. 10.31117/neuroscirn.v8i1.352

[140] Han X, Yuan T, Zhang J et al (2022) FOXO4 peptide targets myofibroblast ameliorates bleomycin-induced pulmonary fibrosis in mice through ECM-receptor interaction pathway. J Cell Mol Med 26:3269–3280. 10.1111/jcmm.17333 35510614 10.1111/jcmm.17333 PMC9170815

[141] Hubackova S, Davidova E, Rohlenova K et al (2019) Selective elimination of senescent cells by mitochondrial targeting is regulated by ANT2. Cell Death Differ 26:276–290. 10.1038/s41418-018-0118-3 29786070 10.1038/s41418-018-0118-3 PMC6329828

[142] Kasperova BJ, Cinkajzlova A, Horvath L et al (2025) Coming of age: could obesity-related metabolic complications be treated by targeting senescent cells? Front Cell Dev Biol. 10.3389/fcell.2025.1622107 40535573 10.3389/fcell.2025.1622107 PMC12174156

[143] Liu S, Xi Q, Li X, Liu H (2025) Mitochondrial dysfunction and alveolar type II epithelial cell senescence: the destroyer and rescuer of idiopathic pulmonary fibrosis. Front Cell Dev Biol. 10.3389/fcell.2025.1535601 40230412 10.3389/fcell.2025.1535601 PMC11994736

[144] Flores NG, Fernández-Aroca DM, Garnés-García C et al (2025) The CDK12–BRCA1 signaling axis mediates dinaciclib-associated radiosensitivity through p53-mediated cellular senescence. Mol Oncol 19:1265–1280. 10.1002/1878-0261.13773 39626031 10.1002/1878-0261.13773 PMC11977655

[145] Younis RL, El-Gohary RM, Ghalwash AA et al (2024) Luteolin mitigates D-galactose-induced brain ageing in rats: SIRT1-mediated neuroprotection. Neurochem Res 49:2803–2820. 10.1007/s11064-024-04203-y 38987448 10.1007/s11064-024-04203-y PMC11365848

[146] Wang J, Lu Y, Carr C et al (2023) Senolytic therapy is neuroprotective and improves functional outcome long-term after traumatic brain injury in mice. Front Neurosci. 10.3389/fnins.2023.1227705 37575310 10.3389/fnins.2023.1227705 PMC10416099

[147] Xu L, Hao L-P, Yu J et al (2023) Curcumin protects against rotenone-induced Parkinson’s disease in mice by inhibiting microglial NLRP3 inflammasome activation and alleviating mitochondrial dysfunction. Heliyon. 10.1016/j.heliyon.2023.e16195 37234646 10.1016/j.heliyon.2023.e16195 PMC10208821

[148] Zhao R, Xu X, Xu F et al (2014) Metformin protects against seizures, learning and memory impairments and oxidative damage induced by pentylenetetrazole-induced kindling in mice. Biochem Biophys Res Commun 448:414–417. 10.1016/j.bbrc.2014.04.130 24802403 10.1016/j.bbrc.2014.04.130

[149] Zhang L-F, Yu X-L, Ji M et al (2018) Resveratrol alleviates motor and cognitive deficits and neuropathology in the A53T α-synuclein mouse model of Parkinson’s disease. Food Funct 9:6414–6426. 10.1039/c8fo00964c 30462117 10.1039/c8fo00964c

[150] Drake SS, Zaman A, Gianfelice C, et al (2024) Senolytic treatment depletes microglia and decreases severity of experimental autoimmune encephalomyelitis. 2024.02.05.579017 10.1186/s12974-024-03278-2 PMC11529445 39487537

[151] Tokui K, Adachi H, Waza M et al (2009) 17-DMAG ameliorates polyglutamine-mediated motor neuron degeneration through well-preserved proteasome function in an SBMA model mouse. Hum Mol Genet 18:898–910. 10.1093/hmg/ddn419 19066230 10.1093/hmg/ddn419

[152] Maximova A, Werry EL, Kassiou M (2021) Senolytics: a novel strategy for neuroprotection in ALS? Int J Mol Sci 22:12078. 10.3390/ijms222112078 34769512 10.3390/ijms222112078 PMC8584291

[153] Han X, Zhang T, Liu H et al (2020) Astrocyte senescence and Alzheimer’s disease: a review. Front Aging Neurosci 12:148. 10.3389/fnagi.2020.00148 32581763 10.3389/fnagi.2020.00148 PMC7297132

[154] Lim S, An SB, Jung M et al (2022) Local delivery of senolytic drug inhibits intervertebral disc degeneration and restores intervertebral disc structure. Adv Healthc Mater 11:e2101483. 10.1002/adhm.202101483 34699690 10.1002/adhm.202101483

[155] Lee J-R, Park B-W, Park J-H et al (2021) Local delivery of a senolytic drug in ischemia and reperfusion-injured heart attenuates cardiac remodeling and restores impaired cardiac function. Acta Biomater 135:520–533. 10.1016/j.actbio.2021.08.028 34454081 10.1016/j.actbio.2021.08.028

[156] Galiana I, Lozano-Torres B, Sancho M et al (2020) Preclinical antitumor efficacy of senescence-inducing chemotherapy combined with a nanoSenolytic. J Control Release 323:624–634. 10.1016/j.jconrel.2020.04.045 32376460 10.1016/j.jconrel.2020.04.045

[157] Pham LM, Kim E-C, Ou W et al (2021) Targeting and clearance of senescent foamy macrophages and senescent endothelial cells by antibody-functionalized mesoporous silica nanoparticles for alleviating aorta atherosclerosis. Biomaterials 269:120677. 10.1016/j.biomaterials.2021.120677 33503557 10.1016/j.biomaterials.2021.120677

[158] Basisty N, Kale A, Jeon OH et al (2020) A proteomic atlas of senescence-associated secretomes for aging biomarker development. PLoS Biol 18:e3000599. 10.1371/journal.pbio.3000599 31945054 10.1371/journal.pbio.3000599 PMC6964821

[159] Mason KD, Carpinelli MR, Fletcher JI et al (2007) Programmed a nuclear cell death delimits platelet life span. Cell 128:1173–1186. 10.1016/j.cell.2007.01.037 17382885 10.1016/j.cell.2007.01.037

